# Mitogenome of the extinct Desert ‘rat-kangaroo’ times the adaptation to aridity in macropodoids

**DOI:** 10.1038/s41598-022-09568-0

**Published:** 2022-04-06

**Authors:** Michael Westerman, Stella Loke, Mun Hua Tan, Benjamin P. Kear

**Affiliations:** 1 Department of Ecology, Environment and Evolution, La Trobe University, Bundoora, VIC 3086 Australia; 2 Deakin Genomics Centre, School of Life and Environmental Sciences, Deakin University, Burwood, VIC 3125 Australia; 3 Department of Microbiology and Immunology, Bio21 Institute, School of Biosciences, University of Melbourne, Melbourne, VIC 3052 Australia; 4 Museum of Evolution, Uppsala University, 752 36 Uppsala, Sweden

**Keywords:** Evolutionary genetics, Phylogenetics, Zoology

## Abstract

The evolution of Australia’s distinctive marsupial fauna has long been linked to the onset of continent-wide aridity. However, how this profound climate change event affected the diversification of extant lineages is still hotly debated. Here, we assemble a DNA sequence dataset of Macropodoidea—the clade comprising kangaroos and their relatives—that incorporates a complete mitogenome for the Desert ‘rat-kangaroo’, *Caloprymnus campestris*. This enigmatic species went extinct nearly 90 years ago and is known from a handful of museum specimens. *Caloprymnus* is significant because it was the only macropodoid restricted to extreme desert environments, and therefore calibrates the group’s specialisation for increasingly arid conditions. Our robustly supported phylogenies nest *Caloprymnus* amongst the bettongs *Aepyprymnus* and *Bettongia*. Dated ancestral range estimations further reveal that the *Caloprymnus*-*Bettongia* lineage originated in nascent xeric settings during the middle to late Miocene, ~ 12 million years ago (Ma), but subsequently radiated into fragmenting mesic habitats after the Pliocene to mid-Pleistocene. This timeframe parallels the ancestral divergences of kangaroos in woodlands and forests, but predates their adaptive dispersal into proliferating dry shrublands and grasslands from the late Miocene to mid-Pleistocene, after ~ 7 Ma. We thus demonstrate that protracted changes in both climate and vegetation likely staged the emergence of modern arid zone macropodoids.

## Introduction

Arid zone marsupials are icons of Australia and have an inferred evolutionary history that extends back over some ~ 15 Ma^1^. Nevertheless, the precise divergence timings of the major extant clades are ambiguous, as are the possible drivers behind their adaptive radiations^2–13^.

**Figure 1:**
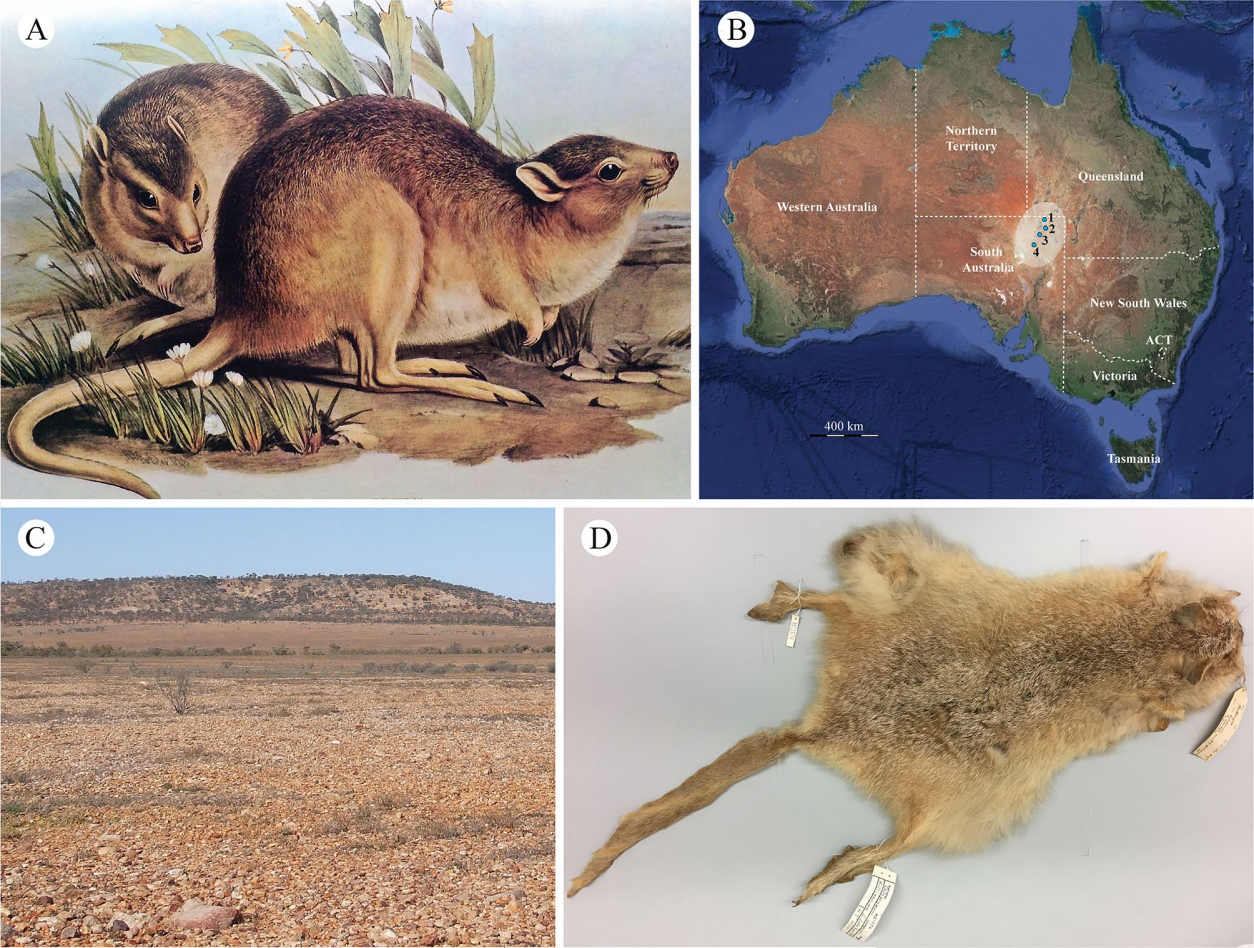
(**A**) Painting of *Caloprymnus campestris* as illustrated by Gould^81^ (image in public domain). (**B**) Estimated historical distribution of *C. campestris* (grey shaded area) and localities from which specimens were collected: (1) Koonchera; (2) Ooroowillanie; (3) Mulka; (4) Killalpaninna (based on data from *Google Maps* and *OZCAM Online Zoological Collections of Australian Museums*: https://ozcam.org.au/). (**C**) Sturt Stony Desert gibber plain habitat of *C. campestris* showing a ‘jump-up’ escarpment and ephemeral drainage channel lined by riparian vegetation in the distance (photograph reproduced with permission from Michael Letnic, University of New South Wales). (**D**) Preserved skin of Caloprymnus campestris (Australian Museum, Sydney [AM] M21674) from Killalpaninna in northeastern South Australia^22^ (photograph reproduced with permission from Mark Eldridge, AM).

Macropodoids (Macropodiformes: Macropodoidea)—the group encompassing living kangaroos, wallaroos, wallabies, pademelons and tree-kangaroos (Macropodidae), bettongs and potoroos (Potoroidae), the Musky rat-kangaroo (*Hypsiprymnodon moschatus*: Hypsyprymnodontidae), and their stem antecedents^14^—incorporate some of the most distinctive Australian arid zone marsupials, as epitomised by the famous Red kangaroo, *Osphranter rufus*^15^. The well-documented fossil record of this and other ‘true kangaroos’ (Macropodini) has been used to correlate arid zone macropodoid evolution with the expansion of intracontinental grasslands during the Pliocene and Pleistocene, from ~ 3–4 Ma^3,9,12^. By contrast, the contemporary diversification of xeric-adapted bettongs is often overlooked, but has considerable significance because it includes the only example of an exclusively desert-inhabiting macropodoid, the Desert ‘rat-kangaroo’, which is alternatively referred to as the “Oolacunta”^16^ or Ngudlukanta^17^, *Caloprymnus campestris* (Fig. 1A).

The first scientific specimens of *C. campestris* were collected from northeastern South Australia (Fig. 1B) in 1842, with three preserved examples subsequently shipped to London for study^18^. These were dubbed ‘*Bettongia*’ *campestris* by Gould^19^, although Thomas^20^ later recognised ‘*B.*’ *campestris* as morphologically distinct from *Bettongia*, and thus established a separate genus, *Caloprymnus*. No further sightings of *C. campestris* were reported after this initial description, and the species was assumed to be extinct for some 90 years until Finlayson^16,21^ announced the “Rediscovery of *Caloprymnus campestris*” in 1931–1932, from the remote Kooncheera Dune^17^ region in the Sturt Stony Desert of far northeastern South Australia (Fig. 1C). Since then, only a skin recovered sometime between 1902 and 1905 (Fig. 1D) has been reidentified^22^, and various unsubstantiated live sightings made^17,23,24^, with the most recent in 2011^24^ and 2013^17^ prompting unsuccessful surveys for the species in 2018 and 2019^17^. *Caloprymnus campestris* has otherwise been classified as Extinct by the IUCN (https://www.iucnredlist.org/) since 1994, with the probable cause being over-predation by feral dogs, cats and foxes^25^.

At latest count, only 25 specimens of *C. campestris* are catalogued in museums worldwide^22^. This dearth of research material has led to uncertainty about potoroid interrelationships^26^, as well as the concomitant chronicle of their arid zone evolution. Here, we therefore analyse the first complete mitochondrial (mt) genome of *C. campestris*, which augments the 12S rRNA (AY245615) and partial cytochrome *b* (AY237246) gene sequences^27^ already available from *GenBank* (https://www.ncbi.nlm.nih.gov/genbank/). Our novel dataset is used to construct a comprehensive phylogeny of crown potoroid species and subspecies within Macropodoidea. We also apply molecular clock calibrated ancestral range estimations to infer both the timing and context of macropodoid habitat change over the last ~ 25 Ma.

## Materials and methods

### Samples and sequencing

We obtained non-formalin-fixed liver samples from a male *Caloprymnus campestris* (Museums Victoria, Melbourne, Australia [NMV] C8981) that was collected in 1834 from Mulka cattle station in northeastern South Australia (Fig. 1B). Our DNA extraction, PCR amplifications, sequencing and alignment procedures followed Westerman et al.^27,28^. Whole genome libraries were prepared with the *Nextera* DNA flex library kit (Illumina, CA), incorporating 50 ng of input DNA per sample. Sequencing was performed on the *Illumina MiSeq* platform using 2 × 300 bp V3 chemistry to generate 4,445,476 read pairs and 1.55 Gb total sequence data. Raw reads were trimmed for adapters and quality using *Trimmomatic* 0.36^29^ (sliding window = 4:15; leading = 3; trailing = 3), and then assembled via genome skimming with *IDBA-UD* 1.1.1^30^ (mink = 20; maxk = 300; min_contig = 500); this yielded an average depth-of-coverage of 76.7x (median = 70x; minimum = 14x; maximum = 311x) and insert length of 109.9 bp. The resulting *C. campestris* mitogenome (A = 34%; C = 24.1%; G = 12.1%; T = 29.8%) was annotated using the *MITOS* webserver^31^ with start-stop positions for protein coding genes manually curated using *blastp* homologies extracted from the NCBI non-redundant (*nr*) database.

### Phylogenetic and molecular clock analyses

Phylogenetic relationships within Macropodoidea were examined using a mitogenome dataset including representatives of all potoroid species, together with *Hypsiprymnodon moschatus* and multiple species-level exemplars for selected macropodid genera (see Supplementary Table S1). The Northern common cuscus, *Phalanger orientalis* (Phalangeridae), and Western pygmy possum, *Cercartetus concinnus* (Burramyidae), were added as non-macropodoid outgroups. To accommodate for recognised gene incongruence^32^, we then compared these results with analyses of nuclear (n), and combined mitogenome/mtDNA/nDNA sequence datasets derived from *GenBank*, which integrated an expanded taxon sample of all potoroid species and subspecies (see Supplementary Tables S1 and S2). The mitogenomes were treated as a single partition, or alternatively sub-partitioned into 12S/16S rRNA stems and loops, pooled 1^st^, 2^nd^ and 3^rd^ protein codon positions, and 3^rd^ codon positions with RY coding to allow for heterogeneity and saturation. A General Time Reversible gene partition model, *gamma* distribution and variable site proportions were determined using *jModelTest*^33^ (Supplementary Table S3).

Tree building employed Maximum likelihood and Bayesian methods implemented in *RAxML* 7.2.8^34^, *MrBayes* 3.2.7^35^ and *BEAST* 2.2.1^36^ with node support calculations based on 1000 bootstrap pseudoreplicates (%) and Bayesian Posterior Probabilities (BPP), respectively. Maximum likelihood used a GTR + I + Γ partition model, while non-dated Bayesian MCMC analyses were run for 6 × 10^6^ generations with a sample frequency of 1000, eight chains, default temperature of 0.2, and burn-in fixed at 6 × 10^4^. Time-trees were constructed in *BEAST* 2.2.1^36^ with relaxed clocks and the minimum–maximum node age constraints listed in the Supplementary Information. Up to 95% of the normal prior distributions were assigned to the interval between minimum and maximum, with 2.5% to each tail. *Gamma* priors (shape = 1; scale = 1) were assigned to the “ucld.mean” parameter for each partition. MCMC analyses were run for 65 × 10^6^ generations with a burn-in of 10 × 10^6^ generations and sampling every 10 × 10^3^ generations. ESS values were > 200 for all estimated parameters. *TreeAnnotator* 2.2.1 (https://www.beast2.org/treeannotator/) was used to summarise the tree sample with mean node heights.

### Ancestral area analyses

Distributional areas were optimised onto the time-calibrated *BEAST* consensus tree and analysed using the *R* package *BioGeoBEARS*^37^ to compare alternative biogeographical range models, and a Bayesian Binary MCMC (BBM) approach^38,39^ to reconstruct ancestral ranges in *RASP* 4^40^. Area codes (Supplementary Table S4) followed standard units^6^ but were refined to represent a generalised vegetation map^41^: A = humid forest (rainforest and/or ‘wet’ sclerophyll dominant) prevalent throughout eastern coastal Australia, western Tasmania and New Guinea; B = woodland (‘dry’ sclerophyll dominant) prevalent throughout northern, eastern and southwestern inland Australia and northeastern Tasmania; C = shrubland (*Acacia* and chenopodiaceous shrubland dominant) prevalent throughout central and central-western Australia; and D = grassland-desert (arid grasslands and/or desert dominant) prevalent in central and central-northwestern Australia. The maximum number of ancestral areas was restricted to three because this equalled the maximum number of areas occupied by our terminal taxa at any given node.

*BioGeoBEARS* comparisons proceeded with likelihood ratio testing of ‘Jumping dispersal events (+ J)’, which have been considered inappropriate for dispersal-extinction-cladogenesis (DEC) models^42^. However, the three parameter Bayesian inference of historical biogeography for discrete areas (BAYAREALIKE) + J model (*P* = 0.0006) received overwhelmingly highest support (AICc = 199.6; AICc_wt = 0.98) for conferring best statistical likelihood on our data (Supplementary Table S5). Finally, we accommodated for connectivity by designating a dispersal multiplier of ‘1’ for adjacent areas (A-B-C)^41^ versus non-adjacent areas (A-D)^41^, which were assigned a value of ‘0.5’.

Our BBM analyses utilised 10 MCMC chains with default temperature 0.1, and run over 5 × 10^6^ generations with sampling frequency and burn-in fixed at 1000. Model settings included ‘Gamma(+ G)’ for among-site rate variation, and ‘Fixed (JC)’ for state frequencies.

## Results and discussion

The *Caloprymnus campestris* mitogenome (16,866 bp) is ordered with 13 protein-coding genes, two ribosomal (r)RNA genes, 21 transfer (t)RNAs, and a non-coding AT-rich control region, which follows the typical configuration for marsupials^43,44^. The tRNAs are arranged around the origin of the L strand (A-C-W-O_L_-N-Y) and intersected between the NADH2 and COX1 genes. Substitution of the anticodon GCC for tRNA^ASP^ (*trnD*) is also consistent with RNA-editing^45^.

Maximum likelihood and Bayesian analyses of our mitogenome dataset produce unanimous resolution of Macropodoidea with Potoroidae as the sister to Macropodidae (Supplementary Figures S1–S6). This pivotal higher-level grouping accords with other crown macropodoid phylogenies^12,46–49^, and warrants a new taxonomic definition^50^, which we coin as Macropodia, new clade, herein (Table 1; Supplementary Information). Bootstrap and BPP support is > 90% for almost all constituent nodes except those uniting: (1) the extinct short-faced kangaroo, *Simosthenurus occidentalis*, with the Banded hare-wallaby, *Lagostrophus fasciatus*, as basally branching macropodids (partitioned/non-partitioned bootstrap = 58/63%; *MrBayes* partitioned/non-partitioned BPP = 0.54/0.56; *BEAST* partitioned/non-partitioned BPP = 1/1); (2) the Quokka, *Setonix brachyurus*, with other macropodines (bootstrap = 48/60%; *MrBayes* BPP = 1/1; *BEAST* BPP = 0.63/0.72); (3) grey kangaroos in the genus *Macropus* with *Osphranter rufus* and brush wallabies representing the genus *Notamacropus* (bootstrap = 80/64%; *MrBayes* BPP = 0.99/1; *BEAST* BPP = 0.99/0.96); and (4) *O. rufus* with *Notamacropus* (bootstrap = 58/55%; *MrBayes* BPP = 0.81/1; *BEAST* BPP = 0.72/0.76). As found by previous studies^5,12,27,46–51^, Potoroinae comprises potoroos within the genus *Potorous* and is distinguished from its sister clade, which we designate Bettonginae^52^ to include the Rufous bettong, *Aepyprymnus rufescens*, as the basally branching sister to *C. campestris* and the species of *Bettongia* (Table 1). Alternative monophyly of *C. campestris* with either *A. rufescens*^53,54^, or the species of *Potorous*^27,50^ were tested using topological constraints in PAUP* 4.0b10^55^ (Supplementary Table S6), but decisively rejected (P < 0.0001***). Taxonomically, therefore, we conclude that the original classification of Gould’s Desert ‘bettong’^19^ as generically consistent with *Bettongia* is feasible, but defer any formal nomenclatural amendment pending a detailed morphological re-evaluation.

**Table 1:** Phylogenetic definitions for Macropodiformes, including Macropodia, new clade, and other selected constituent subclades.

Clade	Definition	Type
Macropodiformes	Most inclusive clade including Balbaroo nalima*, *Hypsiprymnodon moschatus*, *Potorous tridactylus* and *Macropus giganteus*, but excluding *Cercartetus concinnus and Phalanger orientalis*	Stem
Balbaridae*	Most inclusive clade including Balbaroo nalima*, but excluding *Hypsiprymnodon moschatus*, *Potorous tridactylus* and *Macropus giganteus*	Stem
Macropodoidea	Least inclusive clade including *Hypsiprymnodon moschatus*, *Potorous tridactylus* and *Macropus giganteus*	Crown
Hypsiprymnodontidae	Most inclusive clade including *Hypsiprymnodon moschatus* and Propleopus oscillans*, but excluding Balbaroo nalima*, *Potorous tridactylus* and *Macropus giganteus*	Stem
Hypsiprymnodontinae	Most inclusive clade including *Hypsiprymnodon moschatus*, but excluding Propleopus oscillans*	Stem
Propleopinae*	Most inclusive clade including Propleopus oscillans*, but excluding *Hypsiprymnodon moschatus*	Stem
**Macropodia, new clade**	Least inclusive clade including *Potorous tridactylus* and *Macropus giganteus*, but excluding *Hypsiprymnodon moschatus*	Crown
Potoroidae	Least inclusive clade including *Potorous tridactylus* and *Aepyprymnus rufescens*, but excluding *Hypsiprymnodon moschatus* and *Macropus giganteus*	Crown
Potoroinae	Least inclusive clade including *Potorous tridactylus*, but excluding *Aepyprymnus rufescens*	Crown
Bettonginae	Least inclusive clade including *Aepyprymnus rufescens*, but excluding *Potorous tridactylus*	Crown
Macropodidae	Most inclusive clade including Simosthenurus occidentalis*, *Lagostrophus fasciatus* and *Macropus giganteus*, but excluding *Potorous tridactylus* and *Hypsiprymnodon moschatus*	Stem
Sthenurinae*	Most inclusive clade including Simosthenurus occidentalis*, but excluding *Lagostrophus fasciatus* and *Macropus giganteus*	Stem
Lagostrophinae	Most inclusive clade including *Lagostrophus fasciatus*, but excluding Simosthenurus occidentalis* and *Macropus giganteus*	Stem
Macropodinae	Most inclusive clade including *Macropus giganteus*, but excluding Simosthenurus occidentalis* and *Lagostrophus fasciatus*	Stem
Dorcopsini	Least inclusive clade including *Dorcopsis hageni*, but excluding *Dendrolagus lumholtzi* and *Macropus giganteus*	Crown
Dendrolagini	Least inclusive clade including *Dendrolagus lumholtzi*, but excluding *Dorcopsis hageni* and *Macropus giganteus*	Crown
Macropodini	Least inclusive clade including *Macropus giganteus*, but excluding *Dorcopsis hageni* and *Dendrolagus lumholtzi*	Crown

Conceptual explanations and phylogenetic definition registration details are provided in the Supplementary Information.

*Extinct.

**Figure 2:**
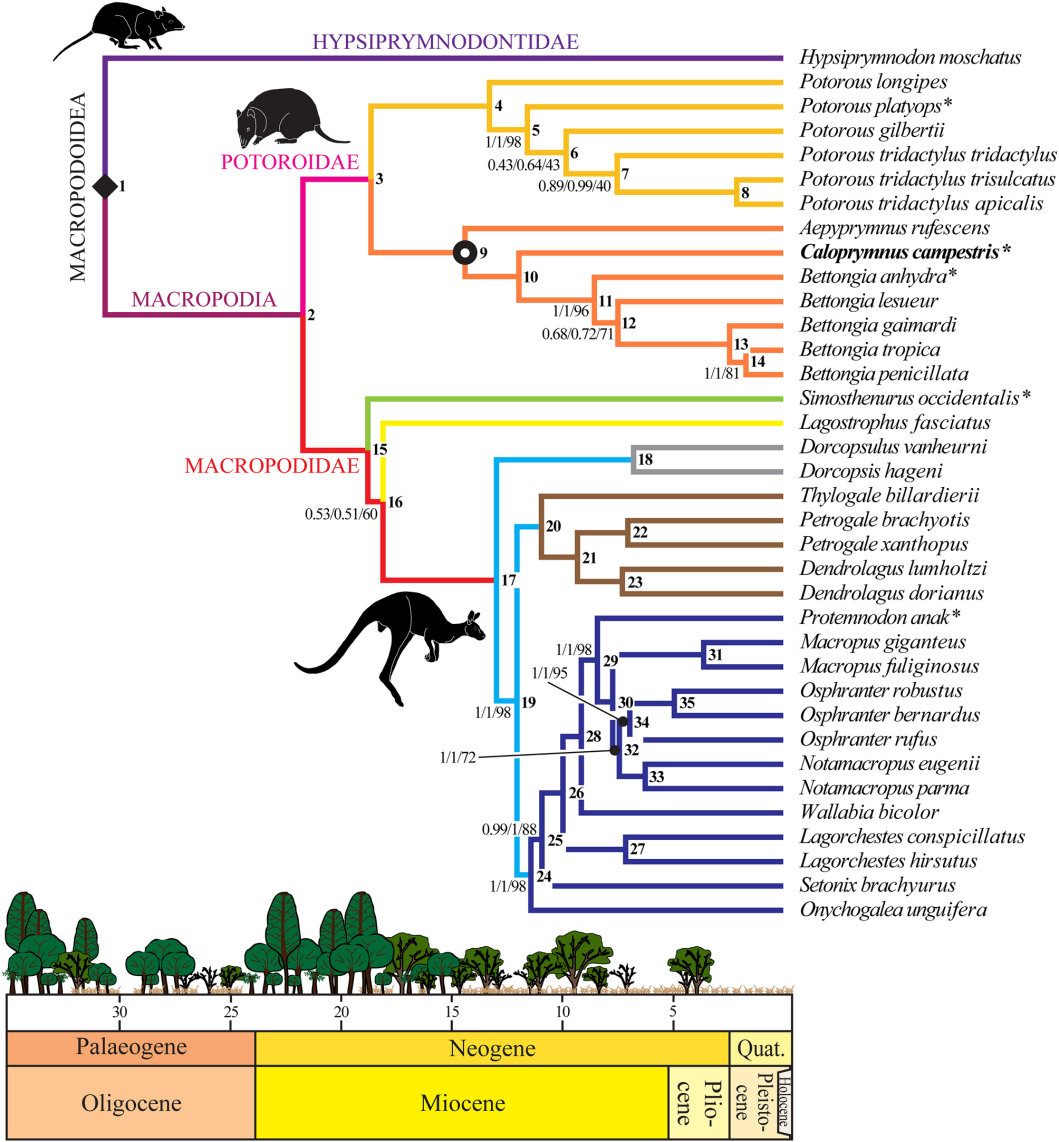
Time calibrated phylogeny of crown Macropodoidea (filled black diamond) showing divergence of *Caloprymnus campestris* (bold type) within Bettonginae (black open circle), and correlated against a schematic of changing palaeohabitats across the late Oligocene–Holocene interval (modified from Kear et al.^6^ and Den Boer et al.^82^). Topology is based on the partitioned mitogenome/mtDNA/nDNA dataset. Bayesian posterior probability (< 1.0) and bootstrap (< 100%) support values (regular type) derived using BEAST 2.2.1^36^/MrBayes 3.2.7^35^/RAxML 7.2.8^34^ are indicated at relevant nodes. Branch colours denote major clades: Hypsiprymnodontidae (purple); Macropodia, new clade (burgundy); Potoroidae (pink); Potoroinae (orange); Bettonginae (ochre); Macropodidae (red); Sthenurinae (green); Lagostrophinae (yellow); Macropodinae (light blue); Dorcopsini (grey) Dendrolagini (brown); Macropodini (dark blue). *Extinct taxa. See Table 2 for node number references (bold type) and the Supplementary Information for other analyses. Graphics produced with Adobe CC2021 by B.P.K.

Our maximum likelihood, Bayesian and time-tree analyses of the nDNA (Supplementary Figures S7–S9) and combined mitogenome/mtDNA/nDNA datasets (Fig. 2; Supplementary Figures S10–S12) yield broadly compatible topologies, with the basal divergence of potoroids and macropodids, and subsequent split between potoroines and bettongines both occurring from the latest Oligocene to earliest-middle Miocene (Table 2; Supplementary Table S7). Notably, this concurs with divergence times derived using different dating methods and constraints^12,46–50,56^. Furthermore, while our *BioGeoBEARS* and BBM ancestral range estimations correlate the latest Eocene (or mid-Eocene using nDNA: Supplementary Table S7) to late Oligocene emergence of crown macropodoids with predominantly humid forest habitats (> 50% probability values from BAYAREALIKE + J [A] = 65.76%; BBM [A] = 61.31%: Supplementary Tables S8 S8 and S9), the initial radiation of potoroids (BAYAREALIKE + J [B/A] = 45.42/25.55%; BBM [B/AB] = 42.62/28.9%), together with the macropodid subclades Sthenurinae (BAYAREALIKE + J [B] = 82.31%; BBM [B] = 66.1%) and Lagostrophinae + Macropodinae (BAYAREALIKE + J [B] = 70.45%; BBM [B/BC] = 41.79/27.29%) are coordinated with earlier Miocene dispersals into woodland dominated mosaics (Fig. 2; Supplementary Tables S8–S11; Supplementary Figures S13 and S14). These potentially included ‘mallee-like’^57^ sclerophyll communities, which propagated throughout central Australia from the early to middle Miocene^41^.

**Table 2:** Estimated divergence times (Ma) with confidence intervals for crown macropodoid clades based on the partitioned mitogenome/mtDNA/nDNA dataset.

Node	Divergence	Time estimate
1	Hypsiprymnodontidae v. Macropodia	30.49 (24.75–36.58)
2	Macropodidae v. Potoroidae	21.91 (18.15–25.66)
3	Potoroinae v. Bettonginae	18.68 (15.36–22.01)
4	*Potorous longipes* v. other *Potorous*	12.71 (10.36–15.23)
5	Potorous platyops* v. other *Potorous*	9.62 (7.57–11.92)
6	*Potorous gilbertii* v. other *Potorous*	8.98 (7.11–11.11)
7	*Potorous tridactylus tridactylus* v. other *Potorous tridactylus* subsp.	7.12 (5.46–8.95)
8	*Potorous tridactylus apicalis* v. *Potorous tridactylus trisulcatus*	2.2 (1.57–2.97)
9	*Aepyprymnus rufescens* v. other Bettonginae	14.62 (11.79–17.4)
10	Caloprymnus campestris* v. *Bettongia* spp.	12.23 (9.77–14.72)
11	Bettongia anhydra* v. other *Bettongia*	8.8 (6.29–11.73)
12	*Bettongia leseuer* v. other *Bettongia*	7.67 (5.86–9.51)
13	*Bettongia gaimardi* v. other *Bettongia*	2.46 (1.81–3.17)
14	*Bettongia tropica* v. *Bettongia penicillata*	1.76 (1.25–2.33)
15	Simosthenurus occidentalis* v. other Macropodidae	19.21 (15.77–22.62)
16	*Lagostrophus fasciatus* v. other Macropodidae	18.6 (15.32–21.9)
17	Dorcopsini v. other Macropodinae	13.54 (11.19–15.96)
18	*Dorcopsulus vanheurni* v. *Dorcopsis hageni*	7.14 (5.41–9.05)
19	Dendrolagini v. Macropodini	12.6 (10.35–14.78)
20	*Thylogale billardierii* v. other Dendrolagini	11.51 (9.55–13.68)
21	*Petrogale* spp. v. *Dendrolagus* spp.	9.89 (8.01–11.69)
22	*Petrogale brachyotis* v. *Petrogale xanthopus*	7.55 (6.04–9.14)
23	*Dendrolagus lumholtzi* v. *Dendrolagus dorianus*	7.79 (6.17–9.42)
24	*Onychogalea unguifera* v. other Macropodini	11.95 (9.81–14.02)
25	*Setonix brachyurus* v. other Macropodini	11.46 (9.47–13.52)
26	*Lagorchestes* spp. v. other Macropodini	10.38 (8.59–12.29)
27	*Lagorchestes hirsutus* v. *Lagorchestes conspicillatus*	7.53 (5.92–9.12)
28	*Wallabia bicolor* v. other Macropodini	9.52 (7.86–11.25)
29	Protemnodon anak* v. other Macropodini	8.84 (7.3–10.44)
30	*Macropus* spp. v. other Macropodini	8.11 (6.68–9.58)
31	*Macropus giganteus* v. *Macropus fuliginosus*	3.84 (2.81–4.94)
32	*Notamacropus* spp. v. *Osphranter* spp.	7.81 (6.43–9.25)
33	*Notamacropus eugenii* v. *Notamacropus parma*	6.57 (5.29–7.9)
34	*Osphranter rufus* v. other *Osphranter* spp.	7.33 (6.01–8.7)
35	*Osphranter robustus* v. *Osphranter bernardus*	5.22 (4.11–6.37)

See Fig. 2 for node number references and the Supplementary Information for other dating analyses.

*Extinct.

The globally recognised^58^ middle to late Miocene climatic transition from equable to increasingly cool, dry conditions^41,59^ coincides with potoroine speciations into mesic environments throughout southern Australia^27,56^. These are tracked by our *BioGeoBEARS* and BBM estimates, which infer occupation of primarily woodland and forest habitats after the earliest-late Miocene (Supplementary Tables S7–S9; Supplementary Figures S13 and S14). This is concurrent with the incipient desertification of inland Australia^60^, which may have promoted genetic segregation of the extinct Broad-faced potoroo, *Potorous platyops*, from Gilbert’s potoroo, *Potorous gilbertii*, in central-southern^61^ and southwestern Australia (BAYAREALIKE + J [B] = 79.64%; BBM [B/AB] = 45.87/44.42%), versus the Long-nosed potoroo, *Potorous tridactylus* (BAYAREALIKE + J [AB] = 77.96%; BBM [AB] = 94.82%), and basally branching Long-footed potoroo, *Potorous longipes*, in southeastern Australia^56^. Additionally, we show that regional subspecies distinctions within *P. tridactylus* were completed by the latest Pliocene to mid-Pleistocene (Table 2; Supplementary Table S7). Curiously, though, Cyt *b* K2P variation (Supplementary Table S12) implies substantially less genetic difference between the Tasmanian *P. tridactylus apicalis* and northeastern mainland *P. tridactylus tridactylus* (1.93%), in comparison to the southeastern mainland *P. tridactylus trisulcatus* (4.21%). Indeed, these values approximate those contrasting *P. tridactylus tridactylus/P. tridactylus trisulcatus* with *P. gilbertii* (2.69/5%), *P. platyops* (4.1/5%), and *P. longipes* (5.84/5.69%), supporting inferences of cryptic taxa^56^, but in our opinion, only up to species-level.

Despite the currently limited DNA sequence coverage for the extinct Finlayson’s^62^ Desert bettong, *Bettongia anhydra*^63^, we derive unequivocal support (Fig. 2; Supplementary Figures S1–S12) for the monophyly of *Bettongia* spp. (bootstrap =  > 90%; BPP = 1), together with close relationships between the woodland-forest dwelling Eastern bettong, *Bettongia gaimardi*, Northern bettong, *Bettongia tropica*, and Brush-tailed bettong, *Bettongia penicillata penicillata* (bootstrap =  > 99%; BPP = 1). Only a few hundred Cyt *b* (or control region) nucleotides are available for the Woylie, *Bettongia penicillata ogilbyi*^64^. Nevertheless, our *BioGeoBEARS* and BBM estimates suggest a latest middle to probably late Miocene divergence of *B. anhydra* (BAYAREALIKE + J [CD] = 98.54%; BBM [CD] = 55.06%) and the Boodie, *Bettongia lesueur*, (BAYAREALIKE + J [CD] = 98.12%; BBM [BCD] = 81.72%) in xeromorphic habitats (Table 2; Supplementary Tables S7–S9; Supplementary Figures S13 and S14), followed by Pliocene to as recent as mid-Pleistocene radiations of *B. gaimardi* (BAYAREALIKE + J [CD] = 90.65%; BBM [BCD/BC] = 27.79/23.75%) and *B. tropica* + *B. penicillata* subsp. (BAYAREALIKE + J [CD] = 79.96.12%; BBM [BCD/BC] = 28.32/21.98%) coupled with increasing habitat variegation^41^. We correlate this with vicariant ‘reversions’^5^ into eucalypt woodlands and forests^65–67^ (Supplementary Tables S10 and S11), which contracted and fragmented with intensifying aridification over the Pliocene–Pleistocene interval^68^.

*Bettongia* is karyotypically conservative, retaining the 2n = 22 chromosomal number of most macropodoids^69,70^. Conversely, chromosomal fission in *P. longipes* has produced 2n = 24, while fusions (and inversions) in *P. tridactylus* and *P. gilbertii* manifest unusual reductions to 2n = 12♀, 13♂^71^. *Aepyprymnus rufescens*, on the other hand, exhibits a unique karyotypic increase to 2n = 32, which is the highest for any marsupial^71^, and presumably reflects its independent evolution since the later-early to early-late Miocene (nDNA favouring a younger later-middle to early-late Miocene range: Table 2; Supplementary Table S7). Although the chromosomal arrangement of *C. campestris* is unknown, our robustly supported (bootstrap =  > 90%; BPP = 1) earliest-middle to early-late Miocene split from *Bettongia* (Table 2; Supplementary Table S7) suggests a similarly protracted ancestry, yet with genetic differentiation that approaches intrageneric levels within *Bettongia* spp. (Cyt *b* K2P variation being as little as 6.91% compared to *B. penicillata*: Supplementary Table S12). Significantly, our *BioGeoBEARS* (BAYAREALIKE + J [CD] = 87.73%) and BBM ([CD] = 53.41%) estimates correlate the *C. campestris*-*Bettongia* divergence with a seminal invasion of xeric environments (Supplementary Tables S8–S12; Supplementary Figures S13 and S14), perhaps incorporating arid chenopod shrublands that spread across central Australia from the middle to late Miocene^41,57,60^. The coeval radiation of macropodines is otherwise linked to predominantly woodland and forest settings (Table 2; Supplementary Tables S8–S12; Supplementary Figures S13 and S14). This includes dorcopsins (BAYAREALIKE + J [B] = 54.21%, BBM [AB/B] = 38.93/25.2%) and dendrolagins (BAYAREALIKE + J [B/A] = 49.35/34.51%, BBM [AB/ABC] = 47.93/26.27%) diverging coincident with uplift of the New Guinean landmass^3,72,73^, and macropodins which initially diversified in woodland habitats (BAYAREALIKE + J [B] = 95.79%; BBM [B] = 77.19%), but subsequently expanded into open shrublands and eventually grasslands (e.g., *Osphranter rufus*: BAYAREALIKE + J [B] = 51.33%, BBM [BC/BCD] = 31.62/24.84%) after the late Miocene to as recent as Pliocene to mid-Pleistocene (Table 2; Supplementary Table S7), thereby presaging the modern prevalence of grazing kangaroos^9^.

## Conclusions

Our characterisation of the complete mitogenome for *Caloprymnus campestris* provides an ecological diversification timescale for bettongs and potoroos within the context of crown macropodoid evolution. Most importantly, we show that the unambiguously monophyletic *C. campestris*-*Bettongia* lineage probably originated with the onset of increasingly arid intracontinental climates during the middle to late Miocene^41,57–60,74^, corresponding with the deepest divergences of Australia’s arid zone biota around ~ 15 Ma^1^. This contrasts with the largely late Miocene to Pleistocene radiation of kangaroos, whose abundance in modern arid zone habitats has been attributed to grazing adaptations and the spread of grasslands during the Pliocene and Pleistocene^3,9,12^. Clearly, therefore, the appearances of Australia’s distinctive arid zone macropodoids were staged over some ~ 3–6 Ma (based on minimum–maximum confidence interval differences for *C. campestris* versus *Osphranter rufus*: Table 2), and likely occurred in response to a complex interplay of abiotic and biotic drivers involving both climate and vegetation change.

Unfortunately, little is known about the biology of *C. campestris* or other extinct ‘Desert bettongs’, such as *Bettongia anhydra*^63^, and the Nullarbor dwarf bettong^75^, *Bettongia pusilla*^76^. Nonetheless, early eye-witness reports state that *C. campestris* inhabited sparsely vegetated gibber plains^16^. The diet of *C. campestris* is also uncertain^23^, but might have been varied^16,23^ similar to the extant arid zone *Bettongia lesueur*^77^ and *Bettongia penicillata*^64^, which consume a range of plant matter, fungi and insects^78,79^. *Caloprymnus campestris* was thus probably an important ‘ecosystem engineer’^63^ whose tragic loss is compounded by dramatic range reductions and the Near Threatened (*Bettongia gaimardi*, *B. lesueur*, *Potorous tridactylus*), Vulnerable (*Potorous longipes*), Endangered (*Bettongia tropica*), Critically Endangered (*B. penicillata*, *Potorous gilbertii*), or Extinct (*B. anhydra*, *C. campestris*, *Potorous platyops*) IUCN Red listings (https://www.iucnredlist.org/) for 10 out of the 11 named non-fossil crown potoroids. The extinction susceptibility of *C. campestris* was presumably exacerbated by its limited distribution (only four recognised collection^22^, and 13 potential sighting localities^17^ within a ~ 350 km radius) and desert specialisation, which when coupled with habitat modification and the introduction of exotic species via European pastoralism^80^, underscores the extreme conservation sensitivity of Australia’s unique arid zone marsupials and the urgent need to document their now dwindling multi-million-year evolutionary histories.

### Ethical approval and informed consent

No live animal subjects were used for experiments in this study. All extinct animal tissues were obtained and their use approved by the La Trobe University Animal Ethics Committee (AEC). All experiments were performed in accordance with institutional guidelines and regulations.

## Supplementary Information

Supplementary Information.

## Data Availability

Raw FASTQ files for the *Caloprymnus campestris* mitogenome assembly have been uploaded onto the *Mendeley Data* repository (https://data.mendeley.com/) under 10.17632/ft8t7v7gfz.1. The consensus *C. campestris* mitogenome (MT663337) and other macropodoid DNA sequences are available from *GenBank* (Supplementary Tables S1 and S2).
