# Author Correction: Mitogenome of the extinct Desert ‘rat-kangaroo’ times the adaptation to aridity in macropodoids

**DOI:** 10.1038/s41598-023-28609-w

**Published:** 2023-01-27

**Authors:** Michael Westerman, Stella Loke, Mun Hua Tan, Benjamin P. Kear

**Affiliations:** 1 Department of Ecology, Environment and Evolution, La Trobe University, Bundoora, VIC 3086 Australia; 2 Deakin Genomics Centre, School of Life and Environmental Sciences, Deakin University, Burwood, VIC 3125 Australia; 3 Department of Microbiology and Immunology, Bio21 Institute, School of Biosciences, University of Melbourne, Melbourne, VIC 3052 Australia; 4 Museum of Evolution, Uppsala University, 752 36 Uppsala, Sweden

Correction to: *Scientific Reports* https://doi.org/10.1038/s41598-022-09568-0, published online 06 April 2022

The original version of this Article contained errors.

In Table 1, the ‘Potoroinae’ clade was incorrectly given as ‘Potorinae’.

In addition, in the Data availability section, the Genbank accession number was incorrect.

“Raw FASTQ files for the *Caloprymnus campestris* mitogenome assembly have been uploaded onto the *Mendeley Data* repository (https://data.mendeley.com/) under https://doi.org/10.17632/ft8t7v7gfz.1. The consensus C. campestris mitogenome (MT66337) and other macropodoid DNA sequences are available from GenBank (Supplementary Tables S1 and S2).”

now reads:

“Raw FASTQ files for the *Caloprymnus campestris* mitogenome assembly have been uploaded onto the *Mendeley Data* repository (https://data.mendeley.com/) under https://doi.org/10.17632/ft8t7v7gfz.1. The consensus C. campestris mitogenome (MT663337) and other macropodoid DNA sequences are available from GenBank (Supplementary Tables S1 and S2).”

The original Article and accompanying Supplementary Information file have been corrected.

