# Supplementary material for: Mitogenome of the extinct Desert ‘rat-kangaroo’ times the adaptation to aridity in macropodoids
Source: Sci Rep. 2022 Apr 6;12:5829. doi: 10.1038/s41598-022-09568-0 (PMC8987032; doi:10.1038/s41598-022-09568-0)
Supplement: Supplementary file 1 — Supplementary Information. [file 41598_2022_9568_MOESM1_ESM.docx]

Supplementary Information for

**Mitogenome of the extinct Desert ‘rat-kangaroo’ times the adaptation to aridity in macropodoids**

Michael Westerman^1^, Stella Loke^2^, Mun Hua Tan^3^ & Benjamin P. Kear^4^

^1^Department of Ecology, Environment and Evolution, La Trobe University, Bundoora, Victoria 3086, Australia. ^2^Deakin Genomics Centre, School of Life and Environmental Sciences, Deakin University, Burwood, Victoria 3125, Australia. ^3^Department of Microbiology and Immunology, Bio21 Institute, School of Biosciences, University of Melbourne, Melbourne, Victoria 3052, Australia. ^4^Museum of Evolution, Uppsala University, SE-752 36 Uppsala, Sweden.

Correspondence and requests for materials should be addressed to M.W. (email: m.westerman@latrobe.edu.au) and B.P.K. (email: benjamin.kear@em.uu.se)

**Supplementary tables**

**Supplementary Table S1. *GenBank* accession numbers for mitogenome and individual mitochondrial (mtDNA) gene sequences used in the phylogenetic analyses.** Taxon abbreviations: Arf, *Aepyprymnus rufescens*; Bah*, Bettongia anhydra*; Bgm, *Bettongia gaimardi*; Bls, *Bettongia lesueur*; Bpn, *Bettongia penicillata*; Btp, *Bettongia tropica*; Ccn, *Cercaretus concinus*; Ccp*, *Caloprymnus campestris**; Ddo, *Dendrolagus dorianus*; Dhg, *Dorcopsis hageni*; Dlm; *Dendrolagus lumholtzi*; Dvh, *Dorcopsulus vanheurni*; Hms, *Hypsiprymnodon moschatus*; Lcs, *Lagorchestes conspicillatus*; Lhs, *Lagorchestes hirsutus*; Lfs, *Lagostrophus fasciatus*; Mfg, *Macropus fuliginosus*; Mgg, *Macropus giganteus*; Neg, *Notamacropus eugenii*; Npm, *Notamacropus parma*; Obn, *Osphranter bernardus*; Orb, *Osphranter robustus*; Orf, *Osphranter rufus*; Pak*, *Protemnodon anak**; Pby, *Petrogale brachyotis*; Pgb, *Potorous gilberti*; Ppl*, *Potorous platyops**; Plg, *Potorous longipes*; Pot, *Phalanger orientalis*; Pta, *Potorous tridactylus apicalis*; Ptd, *Potorous tridactylus tridactylus*; Pts, *Potorous tridactylus trisulcatus*; Pxt, *Petrogale xanthopus*; Sbr, *Setonix brachyurus*; Soc*, *Simosthenurus occidentalis**; Tbl, *Thylogale billardierii*; Wbc, *Wallabia bicolor*. *Extinct.

| **Taxon** | **mitogenome** | **Cyt *b*** | **12S rRNA** | **16S rRNA** | **NADH1** | **COX1/2** |
| --- | --- | --- | --- | --- | --- | --- |
| **Phalangeridae (outgroup)** | | | | | | |
| Pot | MN380186 | – | – | – | – | – |
| **Burramyidae (outgroup)** | | | | | | |
| Ccn | KJ868105 | – | – | – | – | – |
| **Hypsiprymnodontidae** | | | | | | |
| Hms | KJ868115 | – | – | – | – | – |
| **Potoroidae** | | | | | | |
| Arf | KJ868095 | – | – | – | – | – |
| Ccp* | MT663337 | – | – | – | – | – |
| Bah* | – | KM974728 | – | – | – | – |
| Bgm | – | AY237244 | AY245619 | – | KU507321 | MK202809 |
| Bls | KJ868101 | – | – | – | – | – |
| Bpn | KJ868102 | – | – | – | – | – |
| Btp | – | AY237236 | AY245618 | – | JX104579 | JX111907 |
| Pgb | – | AY247231 | AY245616 | – | JX104577 | JX111905 |
| Plg | KJ868148 | – | – | – | – | – |
| Ppl* | – | AY237247 | AY245621 | – | – | – |
| Pta | NC006524 | – | – | – | – | – |
| Ptd | – | AY237235 | JX104620 | – | JX104566 | JX111894 |
| Pts | – | – | JX104626 | – | JX104573 | JX111901 |
| **Taxon (Continued)** | **mitogenome** | **Cyt *b*** | **12S rRNA** | **16S rRNA** | **NADH1** | **COX1/2** |
| **Macropodidae** | | | | | | |
| Ddo | KJ868110 | – | – | – | – | – |
| Dlm | KJ868111 | – | – | – | – | – |
| Dhg | KJ868112 | – | – | – | – | – |
| Dvh | KJ868113 | – | – | – | – | – |
| Lcs | KY996508 | – | – | – | – | – |
| Lhs | AB241056 | – | – | – | – | – |
| Lfs | NC008447 | – | – | – | – | – |
| Mfg | KY868120 | – | – | – | – | – |
| Mgg | KY996502 | – | – | – | – | – |
| Neg | KJ868119 | – | – | – | – | – |
| Npm | KY996504 | – | – | – | – | – |
| Obn | KY996505 | – | – | – | – | – |
| Onu | KJ868133 | – | – | – | – | – |
| Orb | KY996506 | – | – | – | – | – |
| Orf | KY996501 | – | – | – | – | – |
| Pby | KX868141 | – | – | – | – | – |
| Pxt | KX868141 | – | – | – | – | – |
| Pak* | MK190712 | – | – | – | – | – |
| Sbr | KX868156 | – | – | – | – | – |
| Soc* | MK190713 | – | – | – | – | – |
| Tbl | KJ868162 | – | – | – | – | – |
| Wbc | KX868164 | – | – | – | – | – |

**Supplementary Table S2.** ***GenBank* accession numbers for nuclear (nDNA) gene sequences used in the phylogenetic analyses.** Taxon abbreviations explained in Table S1.

| **Taxon** | **ApoB** | **BRCA1** | **IRBP** | **RAG1** | **vWF** |
| --- | --- | --- | --- | --- | --- |
| **Phalangeridae (outgroup)** | | | | | |
| Pot | AF548431 | AY243449 | AY243436 | AY243393 | AY243410 |
| **Burramyidae (outgroup)** | | | | | |
| Ccn | GU566712 | GU566715 | GU566718 | AY125036 | GU566724 |
| **Hypsiprymnodontidae** | | | | | |
| Hms | FJ603118 | FJ603120 | FJ603129 | JN414879 | FJ603139 |
| **Potoroidae** | | | | | |
| Arf | EU160444 | EU160439 | EU160448 | EU160451 | EU160454 |
| Ccp* | – | – | – | – | – |
| Bah* | – | – | – | – | – |
| Bgm | FJ603114 | FJ603168 | FJ603196 | FJ603223 | FJ603250 |
| Bls | – | – | – | – | – |
| Bpn | FJ603145 | FJ603169 | FJ603197 | FJ603224 | FJ603251 |
| Btp | JX104618 |  |  | JX104593 | JX104618 |
| Pgb | JX104616 | JX104603 |  | JX104591 |  |
| Plg | FJ603163 | FJ603191 | FJ603217 | FJ603245 | FJ603272 |
| Ppl* | – | – | – | – | – |
| Pta | FJ603164 | FJ603192 | FJ603218 | FJ603246 | FJ603273 |
| Ptd | JX104615 | JX104594 | – | JX104580 | – |
| Pts | JX104612 | JX104599 | – | JX104587 | – |
| **Macropodidae** | | | | | |
| Ddo | FJ603146 | FJ603170 | FJ603198 | FJ603222 | FJ603252 |
| Dlm | MK211389 | MH197811 | MK211391 | MK197843 | MK211392 |
| Dhg | MT123555 | MT123559 | – | – | MT123549 |
| Dvh | FJ603147 | FJ603173 | FJ603200 | FJ603227 | FJ603255 |
| Lcs | FJ603148 | FJ603174 | FJ603201 | FJ603228 | FJ603256 |
| Lhs | FJ603142 | FJ603175 | FJ603202 | FJ603229 | FJ603257 |
| Lfs | FJ603149 | FJ603176 | FJ603203 | FJ603230 | FJ603258 |
| **Taxon (Continued)** | **ApoB** | **BRCA1** | **IRBP** | **RAG1** | **vWF** |
| Mfg | FJ603143 | FJ603180 | FJ603207 | FJ603234 | FJ603262 |
| Mgg | FJ603153 | FJ603181 | AJ429135 | FJ603235 | AJ224670 |
| Neg | FJ603152 | FJ603179 | FJ603206 | AY059703 | FJ603261 |
| Npm | FJ603155 | FJ603183 | FJ603209 | FJ603237 | FJ603264 |
| Obn | – | – | – | – | – |
| Onu | FJ603160 | FJ603188 | FJ603214 | FJ603242 | FJ603269 |
| Orb | FJ603157 | FJ603185 | FJ603211 | FJ603239 | FJ603266 |
| Orf | FJ603117 | FJ603121 | FJ603127 | FJ607154 | FJ603138 |
| Pby | – | JQ042190 | – | – | – |
| Pxt | FJ603162 | FJ603190 | FJ603216 | FJ603244 | FJ603271 |
| Pak* | – | – | – | – | – |
| Sbr | FJ603165 | FJ603193 | FJ603219 | FJ603247 | FJ603274 |
| Soc* | – | – | – | – | – |
| Tbl | FJ603166 | – | – | – | – |
| Wbc | FJ603167 | – | FJ603221 | – | FJ603276 |
| Ddo | FJ603146 | FJ603170 | FJ603198 | FJ603222 | FJ603252 |

**Supplementary Table S3.** **Gene partition models used in the phylogenetic analyses.**

| **Partition** | **Model** | **P invar** | **Gamma** |
| --- | --- | --- | --- |
| mitogenome | GTR + I + Γ | 0.53 | 0.97 |
| ApoB | GTR + Γ | – | 0.737 |
| BRCA1 | GTR + Γ | – | 0.788 |
| IRBP | GTR + Γ | – | 0.303 |
| RAG 1 | GTR + Γ | – | 0.239 |
| vWF | GTR + I + Γ | 0.393 | 0.825 |

**Supplementary Table S4. Habitat area codes used in the biogeographical analyses.** Taxon abbreviations are explained in Table S1. Habitat area code designations are explained in the main text. *Extinct. †Inferred from ‘browsing’ palaeoecology^83^. †† Inferred from historical distribution^84,85, 86^

| **Taxon** | **Habitat area** | **Code** | **Reference** |
| --- | --- | --- | --- |
| Pot | Humid forest | A | Flannery^87^; Heinsohn^88^ |
| Ccn | Woodland/shrubland | BC | Harris^89^ |
| Hms | Humid forest | A | Johnson & Strahan^90^; Dennis & Johnson^91^ |
| Arf | Woodland | B | Dennis & Johnson^92^ |
| Ccp* | Shrubland/grassland-desert | CD | Vernes *et al*.^17^ |
| Bah* | Shrubland/grassland-desert | CD | McDowell *et al*.^63^ |
| Bgm | Woodland | B | Taylor^65^ |
| Bls | Woodland/shrubland/grassland-desert | BCD | Burbidge & Short^77^ |
| Bpn | Woodland/shrubland/grassland-desert | BCD | Smith *et al*.^67^ |
| Btp | Humid forest/woodland | AB | Laurance^66^ |
| Pgb | Woodland | B | Sinclair *et al*.^93^ |
| Plg | Humid forest | A | Green *et al*.^94^; Menkhorst & Seebeck^95^ |
| ††Ppl* | Woodland | B | Kitchener & Friend^84^ |
| Pta | Humid forest/woodland | AB | Norton *et al*.^96^; Frankham *et al*.^97^ |
| Ptd | Humid forest/woodland | AB | Norton *et al*.^96^; Frankham *et al*.^97^ |
| Pts | Humid forest/woodland | AB | Norton *et al*.^96^; Frankham *et al*.^97^ |
| Ddo | Humid forest | A | Flannery *et al*.^98^ |
| Dlm | Humid forest | A | Flannery *et al*.^98^ |
| Dhg | Humid forest | A | Flannery^87^ |
| Dvh | Humid forest | A | Flannery^87^ |
| Lcs | Woodland/shrubland/grassland-desert | BCD | Ingleby^99^; Burbidge & Johnson^100^ |
| Lhs | Shrubland/grassland-desert | CD | Short & Turner^101^ |
| ††Lfs | Woodland/shrubland | BC | Helgen & Flannery^85^; Prince & Richards^86^ |
| Mfg | Woodland/shrubland | BC | Coulson^102,103^ |
| Mgg | Humid forest/woodland/shrubland | ABC | Coulson^102,104^ |
| Neg | Woodland | B | Hinds^105^ |
| Npm | Humid forest/woodland | AB | Maynes^106^ |
| Obn | Woodland | B | Press^107^; Tefler & Calaby^108^ |
| Onu | Woodland | B | Ingleby & Gordon^109^ |
| Orb | Woodland/shrubland/grassland-desert | BCD | Press^107^; Clancy & Kroft^110^ |
| Orf | Shrubland/grassland-desert | CD | Freedman *et al*.^15^ |
| Pby | Woodland | B | Tefler & Griffiths^111^ |
| Pxt | Shrubland | C | Copley^112^; Lim & Giles^113^ |
| †Pak* | Woodland | B | Johnson & Prideaux^83^ |
| Sbr | Woodland | B | Hayward^114^ |
| †Soc* | Woodland | B | Johnson & Prideaux^83^ |
| Tbl | Humid forest/woodland | AB | Le Mar & McArthur^115^; Johnson & Rose^116^ |
| Wbc | Humid forest/woodland | AB | Merchant^117^ |

**Supplementary Table S5. *BioGeoBEARS* model comparisons.** Log likelihoods (LnL), Akaike information criterion scores (AICc) and weights (AIC_wt) are shown together with *P*-values derived from likelihood ratio tests between LnL values. Selected model is highlighted in bold type.

| **Model** | **LnL** | **AICc** | **AIC_wt** | ***P*-value** |
| --- | --- | --- | --- | --- |
| DEC | -104.7 | 213.8 | 0.0008 | – |
| DEC+J | -101.6 | 210 | 0.0054 | 0.013 |
| DIVALIKE | -105.9 | 216.2 | 0.0002 | – |
| DIVALIKE+J | -103.6 | 213.8 | 0.0008 | 0.029 |
| BAYAREALIKE | -102.3 | 208.9 | 0.0095 | – |
| **BAYAREALIKE+J** | **-96.45** | **199.6** | **0.98** | **0.0006** |

**Supplementary Table S6.** ***PAUP** 4.0b10^55^ constraint tests for monophyly of *Caloprymnus campestris** (Ccp*) with either *Aepyprymnus rufescens* (Arf)^53,54^, or the species of *Potorous* (Pts)^27,50^.** Abbreviations: Con, constraint; Dif, difference; KH, Kishino-Hasegawa test; lnL, log_n_ likelihood; SH, Shimodaira-Hasegawa test; SH-AU, Shimodaira Approximately Unbiased test; w-SH, weighted Shimodaira-Hasegawa test. *Extinct.

| **Con** | **-lnL** | **Dif** | **KH-sd** | **KH-T** | **KH-P** | **SH** | **w-SH** | **SH-AU** |
| --- | --- | --- | --- | --- | --- | --- | --- | --- |
| Ccp*+Arf | 128651.60066 | 131.89083 | 33.562 | 3.930 | 0.0001*** | 0.0001** | 0.0001** | 0.0001*** |
| Ccp*+Pts | 128971.98974 | 452.27991 | 49.539 | 9.130 | <0.0001*** | <0.000** | <0.000** | <0.000** |

**Supplementary Table S7. Estimated divergence times (Ma) with confidence intervals for crown macropodoid clades.** Results were derived from supplementary analyses using the ^P^partitioned mitogenome/mtDNA/nDNA with ^C^*Wallabia bicolor* constrained^12,46,118,119^, the ^N-P^non-partitioned mitogenome/mtDNA/nDNA, ^P^partitioned versus ^N-P^non-partitioned mitogenome, and the nDNA dataset run separately. See Figure 2 and Table 2 for an explanation of the node numbers: 4† = *Potorous longipes* v. *Potorous tridactylus*; 6† = *Potorous gilberti* v. *P. longipes* + *P. tridactylus*; 6†† = *P. longipes* v. *P. tridactylus*; 12† = *Bettongia lesueur* v. *Bettongia penicillata*; 15† = *Simosthenurus occidentalis** + *Lagostrophus fasciatus* v. other Macropodidae; 16† = *S. occidentalis** v. *Lagostrophus fasciatus*; 17† = Dorcopsini v. Macropodini; 24† = *Onychogalea unguifera* v. other Macropodinae; 30† = *Wallabia bicolor* v. *Osphranter* spp. *+ Notamacropus* spp.; 30†† = *Osphranter robustus + Osphranter bernardus* v. other Macropodini; 30††† = *O. robustus* v. other Macropodini; 30†††† = *O. bernardus* v. other Macropodini; 32† = *Osphranter rufus* v. *Notamacropus* spp. *Extinct.

| **Node** | **^PC^Mitogenome/**  **mtDNA/nDNA** | **^N-P^Mitogenome/**  **mtDNA/nDNA** | **^P^Mitogenome** | **^N-P^Mitogenome** | **nDNA** |
| --- | --- | --- | --- | --- | --- |
| 1 | 31.64 (25.83-38.1) | 30.84 (25-36.9) | 31.35 (24.94-37.67) | 31.77 (24.71-38.52) | 37.98 (29.6-46.42) |
| 2 | 22.42 (18.66-26.34) | 21.87 (18.14-25.74) | 21.92 (17.92-25.79) | 22.07 (17.91-26.28) | 22.9 (16.56-27.33) |
| 3 | 19.19 (15.81-22.64) | 18.69 (15.32-22.18) | 18.08 (14.68-21.6) | 17.78 (13.72-22.36) | 19.03 (15.06-23.05) |
| 4 | 13.24 (10.67-15.96) | 12.73 (10.32-15.33) | – | – | – |
| 4† | – | – | 12.48 (9.61-15.43) | 12.43 (9.05-16.22) | – |
| 5 | 10.4 (8.07-12.92) | 10.03 (7.78-12.5) | – | – | – |
| 6 | 9.43 (7.37-11.6) | 9.1 (7.13-11.15) | – | – | – |
| 6† | – | – | – | – | 11.9 (8.84-14.8) |
| 6†† | – | – | – | – | 8.54 (6.08-10.95) |
| 7 | 7.42 (5.65-9.37) | 7.17 (5.46-8.9) | – | – | 3.86 (2.1-5.8) |
| 8 | 2.27 (1.59-3.05) | 2.21 (1.52-2.97) | – | – | 1.32 (0.3-2.5) |
| 9 | 15 (12.31-18.01) | 14.6 (11.82-17.52) | 14.52 (11.04-18.43) | 14.48 (10.05-19.19) | 10.65 (7.33-13.92) |
| 10 | 12.59 (10.17-15.16) | 12.26 (9.83-14.83) | 12.16 (9.72-14.76) | 11.87 (8.92-15.66) | – |
| 11 | 9.07 (6.66-11.87) | 8.69 (6.25-11.68) | – | – | – |
| 12 | 7.42 (6.12-9.73) | 7.66 (5.9-9.51) | – | – | – |
| 12† | – | – | 6.98 (4.65-9.5) | 6.96 (4.16-9.84) | – |
| 13 | 2.54 (1.84-3.26) | 2.47 (1.81-3.23) | – | – | 3.49 (2.03-5.12) |
| 14 | 1.81 (1.29-2.41) | 1.76 (1.25-2.36) | – | – | 1.5 (0.29-2.88) |
| 15 | 19.64 (16.22-23.21) | 19.17 (15.71-22.66) | – | – | – |
| 15† | – | – | 18.73 (15.16-22.4) | 18.94 (14.85-22.74) | – |
| 16 | 19 (15.8-22.52) | 18.51 (15.29-21.91) | – | – | 19.86 (15.59-24.11) |
| 16† | – | – | 16.83 (14.12-21.47) | 17.61 (14-21.11) | – |
| 17 | 13.8 (11.42-16.26) | 13.52 (11.09-16.1) | 13.41 (10.38-16.46) | 13.04 (10.34-15.76) | – |
| **Node (Continued)** | **^PC^Mitogenome/**  **mtDNA/nDNA** | **^N-P^Mitogenome/**  **mtDNA/nDNA** | **^P^Mitogenome** | **^N-P^Mitogenome** | **nDNA** |
| 17† | – | – | – | – | 10.36 (8.02-12.94) |
| 18 | 7.34 (5.52-9.28) | 7.13 (5.34-9.02) | 6.66 (3.16-9.02) | 6.58 (3.62-8.66) | 3.36 (1.82-5.02) |
| 19 | 12.84 (10.64-15.15) | 12.57 (10.34-14.94) | 12.22 (9.65-14.75) | 12.08 (9.91-14.19) | 11.01 (8.48-13.64) |
| 20 | 11.73 (9.68-13.84) | 11.46 (9.45-13.59) | 11.22 (9.02-13.44) | 11.01 (8.75-13.59) | 8.82 (6.58-11.12) |
| 21 | 10.07 (8.27-11.96) | 9.83 (8-11.73) | 9.42 (7.58-11.23) | 9.29 (7.15-11.79) | 7.4 (5.49-9.35) |
| 22 | 7.94 (6.13-9.28) | 7.5 (5.94-9.15) | 7.04 (5.11-8.92) | 6.8 (5.04-9.12) | 4.66 (2.83-6.62) |
| 23 | 7.68 (6.34-9.62) | 7.73 (6.04-9.55) | 7.46 (5.8-9.06) | 7.04 (5.05-8.71) | 5.05 (3.18-7.08) |
| 24 | 12.19 (10.1-14.38) | 11.92 (9.82-14.1) | 11.29 (9-13.66) | 11.32 (9.29-13.34) | – |
| 24† | – | – | – | – | 11.9 (9.14-14.88) |
| 25 | 11.7 (9.65-13.76) | 11.42 (9.44-13.47) | 10.92 (8.93-12.89) | 10.73 (8.18-13.43) | 9.74 (7.38-12.2) |
| 26 | 10.64 (8.82-12.58) | 10.37 (8.47-12.31) | 9.82 (8.02-11.63) | 9.62 (7.32-12.36) | 9.04 (6.82-11.38) |
| 27 | 7.72 (6.14-9.4) | 7.52 (5.91-9.2) | 7.06 (5.2-9.49) | 7.46 (5.9-9.07) | 4.73 (3.06-6.52) |
| 28 | – | 9.5 (7.73-11.25) | 8.98 (7.34-10.7) | 9.02 (7.37-10.67) | – |
| 29 | 9.23 (7.64-10.92) | 8.81 (7.19-10.41) | 8.04 (5.09-10.37) | 8 (5.28-10.63) | – |
| 30 | 8.7 (7.2-10.3) | 8.1 (6.64-9.6) | 6.94 (5.31-8.83) | 6.97 (5.51-8.4) | 8.18 (6.05-10.42) |
| 30† | 8.59 (7.14-10.2) | – | – | – | 7.39 (5.41-9.47) |
| 30†† | – | – | 7.33 (4.09-9.44) | – | – |
| 30††† | – | – | – | 7.51 (6.09-9.05) | – |
| 30†††† | – | – | – | 7.24 (4.62-9.56) | – |
| 31 | 3.98 (2.95-5.1) | 3.82 (2.77-4.99) | 3.37 (1.16-4.7) | 3.28 (1.08-4.29) | 1.24 (0.46-2.12) |
| 32 | 8.17 (6.71-9.66) | 7.8 (6.35-9.21) | – | – | 6.72 (4.88-8.7) |
| 32† | – | – | 6.55 (2.63-8.61) | 6.43 (2.92-8.49) |  |
| 33 | 6.88 (5.56-8.29) | 6.55 (5.27-7.9) | 5.68 (3.72-7.26) | 5.74 (3.25-7.75) | 4.28 (2.75-5.91) |
| 34 | 7.61 (6.23-9.04) | 7.32 (6-8.73) | – | – | 2.98 (1.57-4.47) |
| 35 | 5.47 (4.29-6.67) | 5.22 (4.08-6.46) | 4.91 (2.1-6.43) | – | – |

**Supplementary Table S8. *BioGeoBEARS* ancestral range estimations for crown macropodoid clades.** Probability (%) results derived using the selected BAYAREALIKE+J model (see Supplementary Table S5) and partitioned mitogenome/mtDNA/nDNA dataset. See Figure 2 and Table 2 for an explanation of the node numbers.

|  | **Ancestral Area** | | | | | | | | |
| --- | --- | --- | --- | --- | --- | --- | --- | --- | --- |
| **Node** | **A** | **AB** | **ABC** | **B** | **BC** | **BCD** | **C** | **CD** | **D** |
| 1 | 65.76 | – | – | 18.55 | – | – | 6.15 | 6.98 | 2.55 |
| 2 | 25.55 | – | – | 45.42 | – | – | 11.64 | 13.62 | 3.77 |
| 3 | 25.97 | – | – | 32.1 | – | – | 17.05 | 18.99 | 5.88 |
| 4 | 55.15 | – | – | 28.86 | – | – | 15.99 | – | – |
| 5 | 17.79 | – | – | 47.24 | – | – | 36.97 | – | – |
| 6 | 20.36 | – | – | 79.64 | – | – | – | – | – |
| 7 | 6.97 | 77.96 | – | 15.08 | – | – | – | – | – |
| 8 | 0.11 | 99.69 | – | 0.2 | – | – | – | – | – |
| 9 | – | – | – | 34.45 | – | – | 10.68 | 46.98 | 7.89 |
| 10 | – | – | – | 0.2 | – | – | 6.35 | 87.73 | 5.71 |
| 11 | – | – | – | 0.27 | – | – | 0.61 | 98.54 | 0.56 |
| 12 | 0.02 | – | – | 1.25 | 0.01 | 0.08 | 0.26 | 98.12 | 0.24 |
| 13 | 0.49 | – | – | 8.76 | – | – | 0.05 | 90.65 | 0.05 |
| 14 | 0.7 | 0.85 | – | 6.08 | 0.38 | 19.16 | 0.24 | 71.96 | 0.25 |
| 15 | 11.92 | – | – | 82.31 | – | – | 5.34 | 0.38 | 0.05 |
| 16 | 17.58 | – | – | 70.45 | 0.81 | – | 10.66 | 0.39 | 0.11 |
| 17 | 41.19 | – | – | 54.21 | 0.04 | – | 3.46 | 0.93 | 0.16 |
| 18 | 1.0 | – | – | – | – | – | – | – | – |
| 19 | 20.85 | – | 0.18 | 71.68 | 0.05 | – | 5.83 | 1.2 | 0.29 |
| 20 | 34.51 | 0.77 | 0.55 | 49.35 | 1.4 | – | 13.42 | – | – |
| 21 | 43.43 | – | – | 39.66 | – | – | 16.91 | – | – |
| 22 | – | – | – | 61.38 | – | – | 38.62 | – | – |
| 23 | 1.0 | – | – | – | – | – | – | – | – |
| 24 | 0.43 | – | – | 95.79 | – | – | 1.3 | 1.62 | 0.87 |
| 25 | 0.68 | – | – | 92 | – | – | 2.61 | 2.49 | 2.19 |
| 26 | 2.22 | 0.13 | – | 72.07 | 2.65 | 0.03 | 8.05 | 10.2 | 7.28 |
| 27 | – | – | – | 9.44 | 2.66 | 2.65 | 8.91 | 67.55 | 8.77 |
| 28 | 5.3 | 0.27 | – | 89.04 | – | – | 1 | 4.14 | 0.23 |
| 29 | 0.17 | – | – | 92.73 | – | – | 2.29 | 4.36 | 0.45 |
| 30 | 0.49 | 0.01 | 0.01 | 84.46 | 0.81 | – | 7.85 | 5.07 | 1.3 |
| 31 | 0.25 | 0.76 | 0.32 | 4.03 | 93.67 | – | 0.96 | – | – |
| 32 | 0.49 | 0.01 | 0.01 | 84.46 | 0.81 | – | 7.85 | 5.07 | 1.3 |
| 33 | 4.81 | – | – | 95.19 | – | – | – | – | – |
| 34 | – | – | – | 51.33 | – | – | 14.72 | 20.32 | 13.62 |
| 35 | – | – | – | 64.07 | – | – | 5.86 | 24.53 | 5.32 |

**Supplementary Table S9. Bayesian Binary MCMC (BBM) ancestral range estimations for crown macropodoid clades.** Probability (%) results combined over two runs using the selected JC+G model and partitioned mitogenome/mtDNA/nDNA dataset. See Figure 2 and Table 2 for an explanation of the node numbers.

|  | **Ancestral Range** | | | | | | | | | | | |
| --- | --- | --- | --- | --- | --- | --- | --- | --- | --- | --- | --- | --- |
| **Node** | **A** | **AB** | **ABC** | **ABD** | **AC** | **B** | **BC** | **BCD** | **BD** | **C** | **CD** | **D** |
| 1 | 61.31 | 20.67 | 1.24 | 0.12 | 3.69 | 9.97 | 0.6 | – | 0.06 | 1.78 | 0.01 | 0.17 |
| 2 | 13.94 | 28.9 | 3.69 | 0.25 | 1.78 | 42.62 | 5.44 | 0.05 | 0.37 | 2.62 | 0.02 | 0.18 |
| 3 | 14.96 | 24.24 | 5.07 | 0.53 | 3.13 | 37.44 | 7.83 | 0.17 | 0.81 | 4.83 | 0.1 | 0.5 |
| 4 | 41.77 | 22.79 | 2.4 | 0.15 | 4.04 | 20.8 | 2.01 | 0.01 | 0.13 | 3.39 | 0.02 | 0.21 |
| 5 | 18.5 | 17.53 | 4.92 | 0.11 | 5.19 | 33.64 | 8.36 | 0.06 | 0.21 | 9.44 | 0.06 | 0.22 |
| 6 | 6.98 | 44.42 | 0.98 | 0.18 | 0.15 | 45.87 | 1.01 | 0.06 | 0.18 | 0.16 | 0.06 | 0.03 |
| 7 | 2.47 | 94.82 | 0.59 | 0.23 | 0.02 | 1.84 | 0.01 | – | – | – | – | – |
| 8 | 0.51 | 98.37 | 0.36 | 0.15 | – | 0.6 | – | – | – | – | – | – |
| 9 | 3.04 | 10.09 | 4.22 | 0.88 | 1.27 | 46.98 | 19.67 | 1.71 | 4.09 | 5.93 | 0.52 | 1.23 |
| 10 | 0.02 | 0.01 | 0.17 | 0.06 | 0.33 | 0.22 | 4.02 | 27.09 | 1.47 | 7.92 | 53.41 | 2.9 |
| 11 | – | – | 0.03 | 0.04 | 0.06 | 0.06 | 1.32 | 36.1 | 1.55 | 2.01 | 55.06 | 2.36 |
| 12 | – | – | 0.24 | 0.22 | 0.02 | 0.39 | 5.9 | 81.72 | 5.37 | 0.37 | 5.18 | 0.34 |
| 13 | 0.06 | 1.97 | 2.59 | 2.3 | 0.08 | 18.03 | 23.75 | 27.79 | 21.1 | 0.71 | 0.83 | 0.63 |
| 14 | 0.14 | 5.11 | 10.23 | 6.58 | 0.27 | 10.97 | 21.98 | 28.32 | 14.14 | 0.59 | 0.76 | – |
| 15 | 0.9 | 16.96 | 7.39 | 3.01 | 0.16 | 66.1 | 11.73 | 0.06 | 0.34 | 0.62 | – | – |
| 16 | 1.31 | 15.96 | 10.56 | 0.09 | 0.86 | 41.25 | 27.29 | 0.16 | 0.23 | 2.23 | 0.01 | – |
| 17 | 11.26 | 38.93 | 6.6 | 11.31 | 3.27 | 25.2 | 7.32 | 0.16 | 0.16 | 2.12 | 0.01 | 0.05 |
| 18 | 91.72 | 5.97 | 0.09 | 0.01 | 1.45 | 0.43 | 0.01 | – | – | 0.1 | – | – |
| 19 | 2.79 | 39.43 | 13.57 | 0.27 | 0.96 | 31.17 | 10.73 | 0.07 | 0.21 | 0.76 | 0.01 | 0.02 |
| 20 | 6.59 | 47.93 | 26.27 | 0.24 | 3.61 | 9.38 | 5.14 | 0.03 | 0.05 | 0.71 | – | 0.01 |
| 21 | 31.24 | 32.74 | 7.67 | 0.21 | 7.32 | 13.96 | 3.27 | 0.02 | 0.09 | 3.12 | 0.02 | 0.09 |
| 22 | 7.53 | 11.63 | 3.56 | 0.06 | 2.31 | 49.37 | 15.12 | 0.08 | 0.27 | 9.8 | 0.05 | 0.17 |
| 23 | 95.13 | 3.07 | 0.04 | 0.01 | 1.26 | 0.2 | – | – | – | 0.08 | – | 0.01 |
| 24 | 0.16 | 13.56 | 1.28 | 0.06 | 0.01 | 77.19 | 7.27 | 0.03 | 0.35 | 0.09 | – | – |
| 25 | 0.18 | 10.13 | 0.93 | 0.11 | 0.02 | 80.16 | 7.35 | 0.08 | 0.9 | 0.13 | – | 0.02 |
| 26 | 0.79 | 18.13 | 6.21 | 1.28 | 0.27 | 50.31 | 17.22 | 1.21 | 3.54 | 0.75 | 0.05 | 0.15 |
| 27 | 0.01 | 0.06 | 0.98 | 0.37 | 0.2 | 0.6 | 10.37 | 66.12 | 3.85 | 2.08 | 13.26 | 0.77 |
| 28 | 0.65 | 48.28 | 7.63 | 0.4 | 0.1 | 36.69 | 5.8 | 0.05 | 0.31 | 0.08 | – | – |
| 29 | 0.28 | 21.51 | 4.21 | 0.12 | 0.05 | 61.28 | 11.99 | 0.07 | 0.33 | 0.16 | – | – |
| 30 | 0.42 | 18.93 | 17.32 | 0.24 | – | 32 | 29.28 | 0.36 | 0.4 | 0.64 | 0.01 | 0.01 |
| 31 | 0.06 | 6.04 | 45.78 | 0.02 | 0.44 | 5.49 | 41.62 | 0.13 | 0.02 | 0.4 | – | – |
| 32 | 0.95 | 19.53 | 13.25 | 1.13 | 0.64 | 35.53 | 24.1 | 1.39 | 2.05 | 1.17 | 0.07 | 0.1 |
| 33 | 1.29 | 48.33 | 2.23 | 0.23 | 0.06 | 45.47 | 2.1 | 0.01 | 0.22 | 0.06 | – | 0.01 |
| 34 | 0.33 | 1.75 | 4.69 | 1.37 | 0.26 | 11.79 | 31.62 | 24.84 | 9.26 | 6.01 | 4.72 | 1.76 |
| 35 | 0.04 | 1.26 | 2.16 | 1.01 | 0.06 | 19.18 | 32.71 | 26.12 | 15.32 | 0.92 | 0.73 | 0.43 |

**Supplementary Table S10. *BioGeoBEARS* dispersal-vicariance-extinction estimations for crown macropodoid clades.** Event matrix derived using the selected BAYAREALIKE+J model (see Supplementary Table S5) and partitioned mitogenome/mtDNA/nDNA dataset. Speciation within areas: A = 6; B = 17; C = 5; D= 4. See Figure 2 and Table 2 for an explanation of the node numbers.

|  | **Event** | | | | |
| --- | --- | --- | --- | --- | --- |
| **Node** | **Dispersal** | **Vicariance** | **Extinction** | **Route** | **Probability** |
| 1 | 1 | 1 | – | A→AB→A\|B | 0.3 |
| 2 | – | – | – | B→B^∧^B→B\|B | 0.12 |
| 3 | 2 | 1 | 1 | B→→ACD→A\|CD | 0.08 |
| 4 | 1 | 1 | – | A→AB→A\|B | 0.26 |
| 5 | 1 | 1 | – | B→BC→C\|B | 0.38 |
| 6 | 1 | – | – | B→ B^∧^B→AB^∧^B→B\|AB | 0.62 |
| 7 | 2 | – | – | AB→AB^∧^A^∧^B→AB\|AB | 0.73 |
| 8 | 2 | – | – | AB→AB^∧^A^∧^B→AB\|AB | 1.0 |
| 9 | 1 | 1 | – | CD→BCD→B\|CD | 0.41 |
| 10 | 2 | – | – | CD→CD^∧^C^∧^D→CD\|CD | 0.86 |
| 11 | 2 | – | – | CD→CD^∧^C^∧^D→CD\|CD | 0.97 |
| 12 | 3 | – | – | CD→CD^∧^C^∧^D→BCD^∧^C^∧^D→CD\|CD | 0.89 |
| 13 | 1 | 1 | – | CD→BCD→B\|CD | 0.65 |
| 14 | 3 | 1 | – | CD→CD^∧^B→ABCD^∧^B→AB\|BCD | 0.72 |
| 15 | – | – | – | B→B^∧^B→B\|B | 0.58 |
| 16 | 1 | – | – | B→B^∧^B→BC^∧^B→BC\|B | 0.38 |
| 17 | 1 | 1 | – | B→AB→B\|A | 0.39 |
| 18 | – | – | – | A→A^∧^A→A\|A | 1.0 |
| 19 | – | – | – | B→B^∧^B→B\|B | 0.34 |
| 20 | 3 | 1 | – | B→B^∧^A→ABC^∧^A→ABC\|A | 0.21 |
| 21 | 1 | 1 | – | A→AB→A\|B | 0.27 |
| 22 | 1 | 1 | – | B→BC→C\|B | 0.61 |
| 23 | – | – | – | A→A^∧^A→A\|A | 1.0 |
| 24 | – | – | – | B→B^∧^B→B\|B | 0.88 |
| 25 | – | – | – | B→B^∧^B→B\|B | 0.66 |
| 26 | 2 | 1 | – | B→BCD→B\|CD | 0.43 |
| 27 | 3 | – | – | CD→CD^∧^C^∧^D→BCD^∧^C^∧^D→CD\|BCD | 0.68 |
| 28 | 1 | – | – | B→B^∧^B→AB^∧^B→AB\|B | 0.83 |
| 29 | – | – | – | B→B^∧^B→B\|B | 0.78 |
| 30 | 1 | – | – | B→B^∧^B→BC^∧^B→B\|BC | 0.64 |
| 31 | 3 | – | – | BC→BC^∧^B^∧^C→ABC^∧^B^∧^C→BC\|ABC | 0.94 |
| 32 | – | – | – | B→B^∧^B→B\|B | 0.39 |
| 33 | 1 | – | – | B→B^∧^B→AB^∧^B→AB\|B | 0.95 |
| 34 | 2 | 1 | – | B→BCD→CD\|B | 0.33 |
| 35 | 2 | – | – | B→B^∧^B→BCD^∧^B→B\|BCD | 0.64 |

**Supplementary Table S11. Bayesian Binary MCMC (BBM) dispersal-vicariance-extinction estimations for crown macropodoid clades.** Event matrix derived using the selected JC+G model and partitioned mitogenome/mtDNA/nDNA dataset. Speciation within areas: A = 7; B = 21; C = 6; D= 4. See Figure 2 and Table 2 for an explanation of the node numbers.

|  | **Event** | | | | |
| --- | --- | --- | --- | --- | --- |
| **Node** | **Dispersal** | **Vicariance** | **Extinction** | **Route** | **Probability** |
| 1 | 1 | 1 | – | A→AB→A\|B | 0.26 |
| 2 | – | – | – | B→B^∧^B→B\|B | 0.11 |
| 3 | 1 | 1 | – | B→→AB→A\|B | 0.07 |
| 4 | 1 | 1 | – | A→AB→A\|B | 0.14 |
| 5 | 1 | 1 | – | B→BC→C\|B | 0.15 |
| 6 | 1 | – | – | B→ B^∧^B→AB^∧^B→B\|AB | 0.43 |
| 7 | 2 | – | – | AB→AB^∧^A^∧^B→AB\|AB | 0.93 |
| 8 | 2 | – | – | AB→AB^∧^A^∧^B→AB\|AB | 0.98 |
| 9 | 2 | 1 | – | CD→BCD→B\|CD | 0.25 |
| 10 | 2 | – | – | CD→CD^∧^C^∧^D→CD\|CD | 0.29 |
| 11 | 3 | – | – | CD→CD^∧^C^∧^D→BCD^∧^C^∧^D→CD\|BCD | 0.45 |
| 12 | 3 | – | – | BCD→BCD^∧^B^∧^C^∧^D→BCD\|BCD | 0.23 |
| 13 | 1 | – | – | BCD→ BCD^∧^B→B\|BCD | 0.08 |
| 14 | 2 | – | – | BCD→BCD^∧^B→ABCD^∧^B→AB\|BCD | 0.28 |
| 15 | – | – | – | B→B^∧^B→B\|B | 0.27 |
| 16 | 2 | – | – | B→B^∧^B→ABC^∧^B→BC\|AB | 0.16 |
| 17 | 1 | – | – | AB→AB^∧^B→B\|AB | 0.15 |
| 18 | – | – | – | A→A^∧^A→A\|A | 0.92 |
| 19 | 1 | – | – | AB→AB^∧^B→B\|AB | 0.15 |
| 20 | 3 | – | – | AB→AB^∧^A^∧^B→ABC^∧^A^∧^B→ABC\|AB | 0.16 |
| 21 | – | 1 | – | AB→A\|B | 0.15 |
| 22 | 1 | 1 | – | B→BC→C\|B | 0.49 |
| 23 | – | – | – | A→A^∧^A→A\|A | 0.95 |
| 24 | – | – | – | B→B^∧^B→B\|B | 0.62 |
| 25 | – | – | – | B→B^∧^B→B\|B | 0.40 |
| 26 | 3 | – | – | B→B^∧^B→ABCD^∧^B→AB\|BCD | 0.16 |
| 27 | 2 | – | – | BCD→BCD^∧^C^∧^D→CD\|BCD | 0.66 |
| 28 | 1 | – | – | AB→AB^∧^B→AB\|B | 0.30 |
| 29 | – | – | – | B→B^∧^B→B\|B | 0.12 |
| 30 | 2 | – | – | B→B^∧^B→ABC^∧^B→B\|ABC | 0.05 |
| 31 | 2 | – | – | ABC→ABC^∧^B^∧^C→BC\|ABC | 0.46 |
| 32 | – | – | – | B→B^∧^B→ABC^∧^B→BC\|AB | 0.05 |
| 33 | 1 | – | – | AB→AB^∧^B→AB\|B | 0.48 |
| 34 | 2 | – | – | BC→BC^∧^C→BCD^∧^C→CD\|BC | 0.1 |
| 35 | 2 | – | – | BC→BC^∧^B→BCD^∧^B→B\|BCD | 0.33 |

**Supplementary Table S12. Comparative Cyt *b* K2P values for potoroid species and subspecies.** Taxon abbreviations are explained in Table S1.

|  | **Pta** | **Ptd** | **Pts** | **Pgb** | **Ppl*** | **Plg** | **Bpn** | **Btp** | **Bgm** | **Bls** | **Ccp*** | **Arf** |
| --- | --- | --- | --- | --- | --- | --- | --- | --- | --- | --- | --- | --- |
| Pta | – | 1.928 | 4.211 | 5.051 | 6.540 | 6.083 | 7.601 | 6.902 | 7.602 | 8.062 | 8.575 | 7.556 |
| Ptd | – | – | 3.540 | 2.693 | 4.089 | 5.843 | 7.713 | 7.028 | 7.603 | 7.932 | 8.432 | 7.990 |
| Pts | – | – | – | 4.998 | 4.998 | 5.685 | 7.270 | 6.736 | 7.266 | 8.128 | 8.248 | 7.298 |
| Pgb | – | – | – | – | 5.730 | 3.589 | 8.823 | 8.023 | 8.244 | 9.301 | 9.424 | 8.729 |
| Ppl* | – | – | – | – | – | 6.540 | 7.463 | 6.663 | 7.358 | 8.288 | 8.431 | 7.869 |
| Plg | – | – | – | – | – | – | 7.728 | 8.004 | 7.740 | 7.353 | 7.961 | 7.291 |
| Bpn | – | – | – | – | – | – | – | 2.485 | 1.712 | 6.885 | 6.906 | 7.231 |
| Btp | – | – | – | – | – | – | – | – | 2.040 | 4.252 | 7.147 | 7.952 |
| Bgm | – | – | – | – | – | – | – | – | – | 4.252 | 7.147 | 7.953 |
| Bls | – | – | – | – | – | – | – | – | – | – | 6.995 | 8.434 |
| Ccp* | – | – | – | – | – | – | – | – | – | – | – | 8.093 |
| Arf | – | – | – | – | – | – | – | – | – | – | – | – |

**Supplementary figures**

**
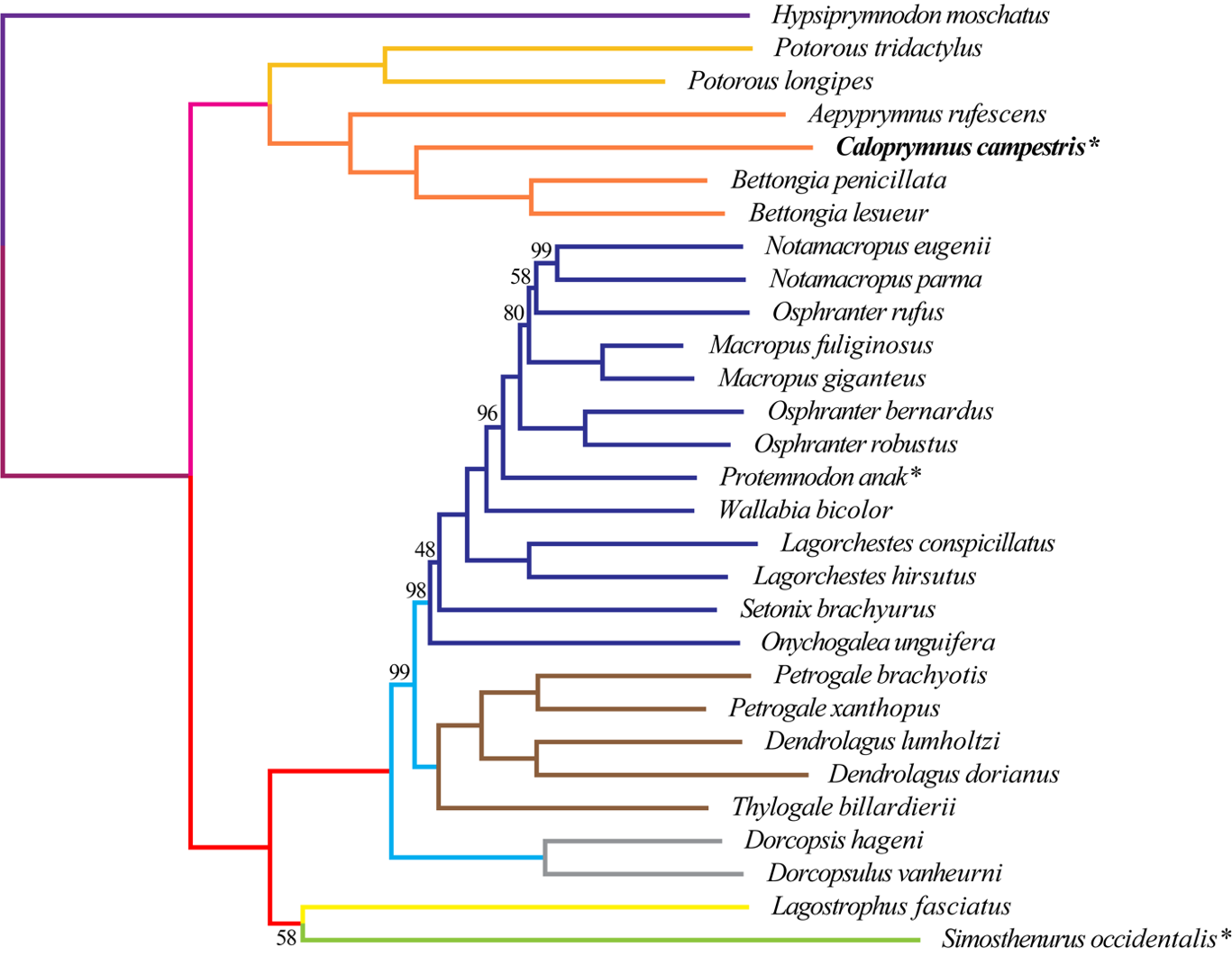
**

**Supplementary Figure S1. Maximum likelihood consensus tree of the partitioned mitogenome dataset generated by *RAxML* 7.2.8^34^.** Bootstrap support values (<100%) are indicated at relevant nodes. Branch colours denote major clades: Hypsiprymnodontidae (purple); Macropodia, new clade (burgundy); Potoroidae (pink); Potoroinae (orange); Bettonginae (ochre); Macropodidae (red); Sthenurinae (green); Lagostrophinae (yellow); Macropodinae (light blue); Dorcopsini (grey) Dendrolagini (brown); Macropodini (dark blue). *Extinct taxa.

**
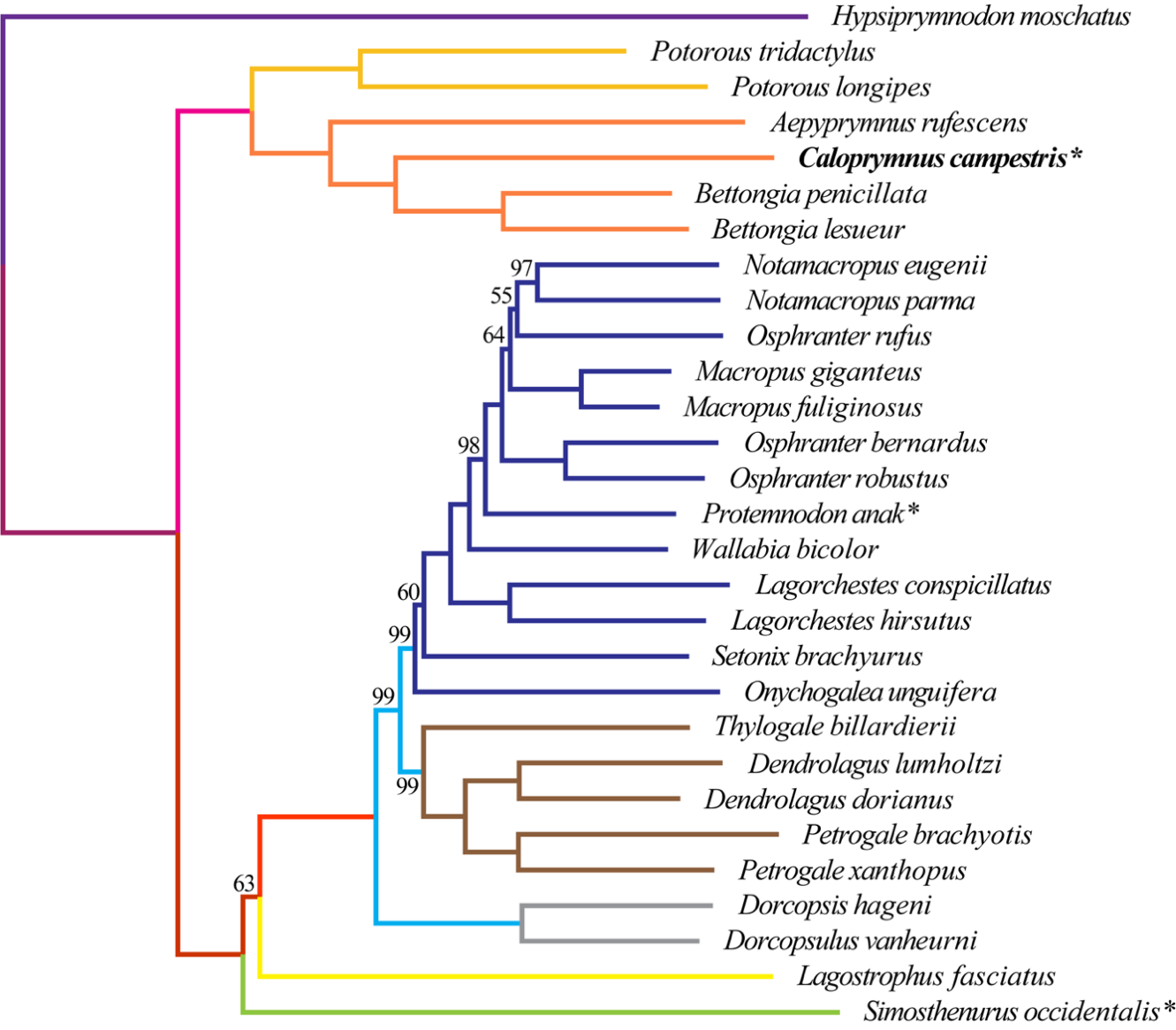
**

**Supplementary Figure S2. Maximum likelihood consensus tree of the non-partitioned mitogenome dataset generated by *RAxML* 7.2.8.** Bootstrap support values (<100%) are indicated at relevant nodes. Branch colours denoting major clades follow Supplementary Figure S1.

**
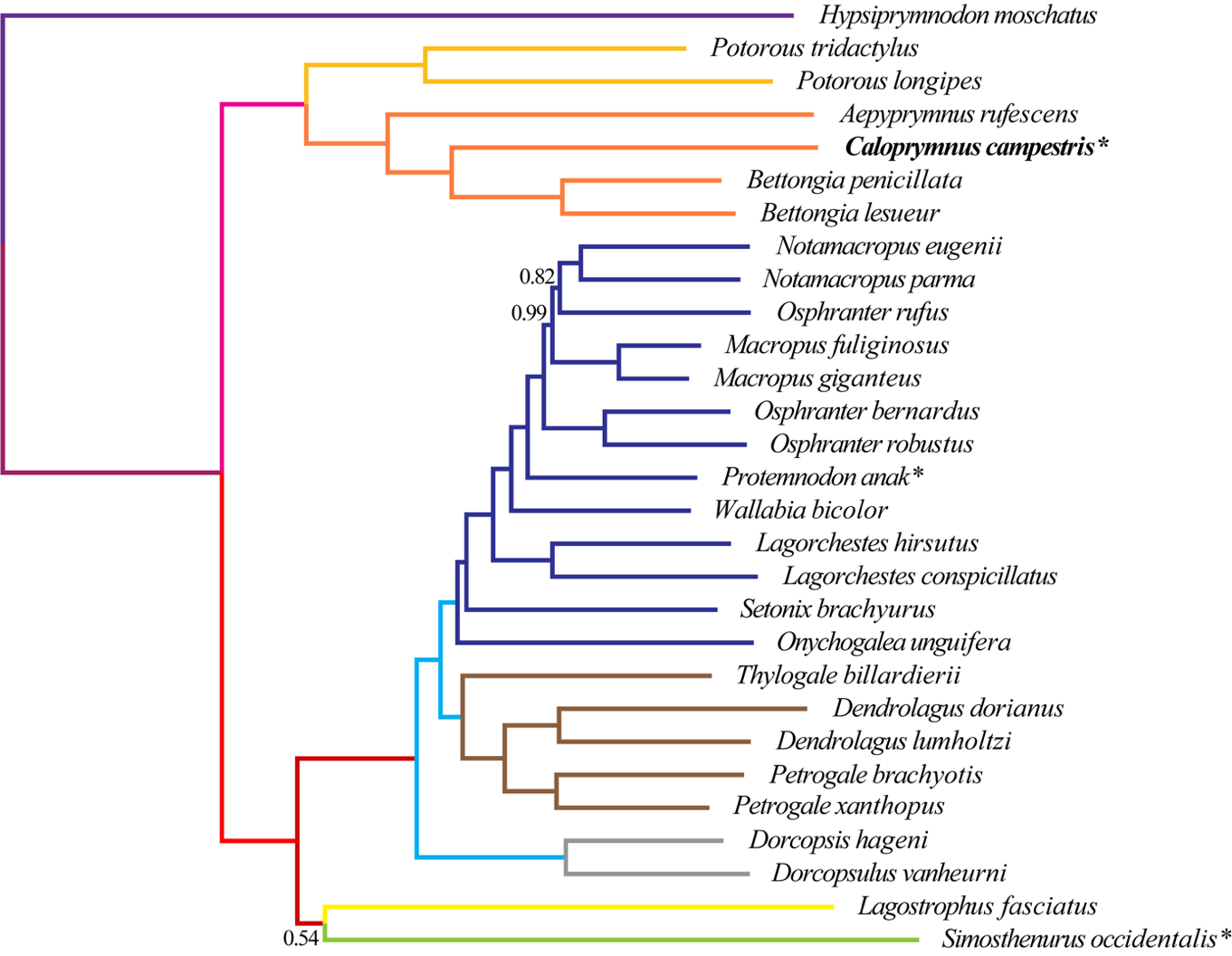
**

**Supplementary Figure S3. Bayesian consensus tree of the partitioned mitogenome dataset generated by *MrBayes* 3.2.7^35^.** Bayesian Posterior Probability (BPP) support values (<1.0) are indicated at relevant nodes. Branch colours denoting major clades follow Supplementary Figure S1.

**
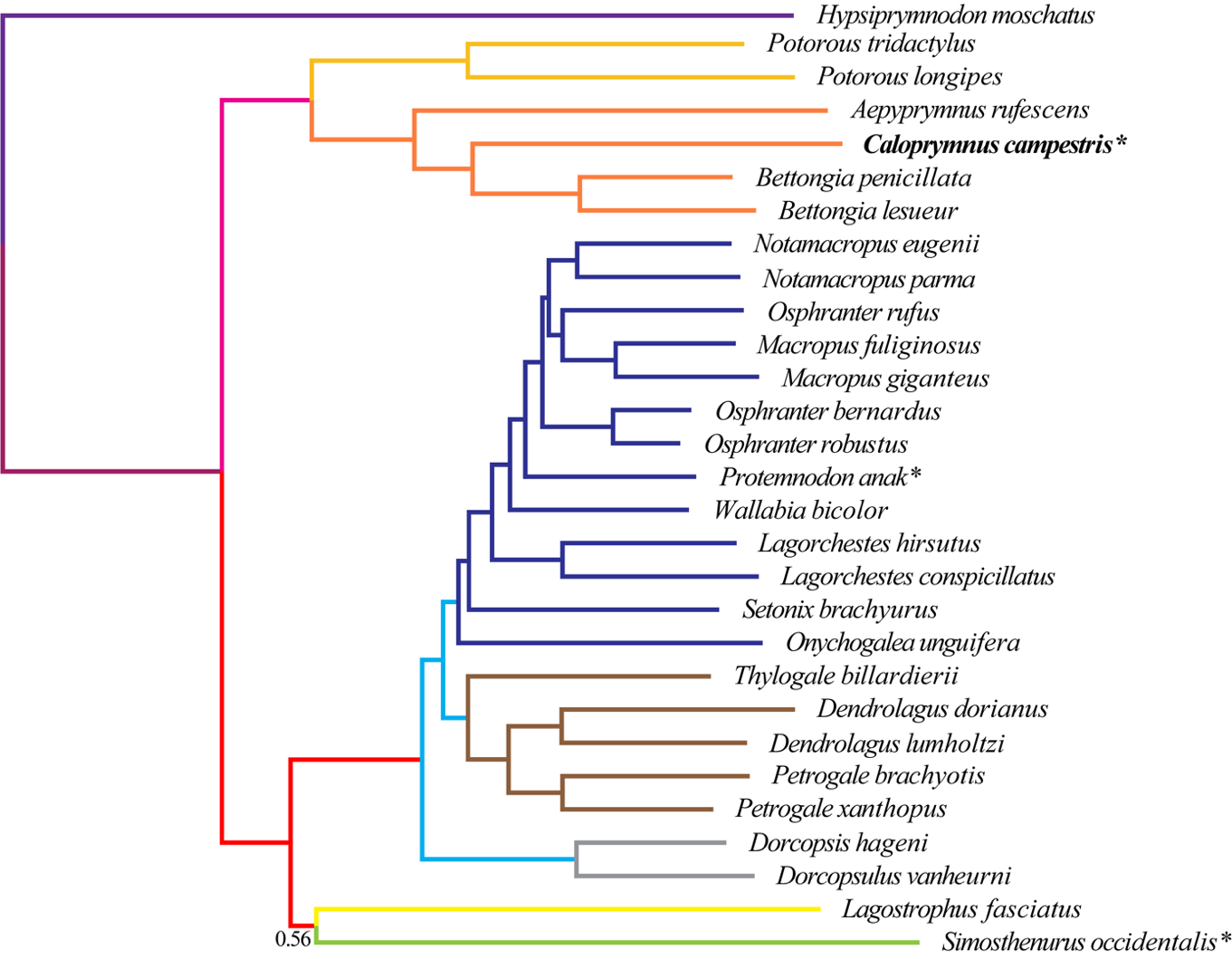
**

**Supplementary Figure S4. Bayesian consensus tree of the non-partitioned mitogenome dataset generated by *MrBayes* 3.2.7.** BPP support values (<1.0) are indicated at relevant nodes. Branch colours denoting major clades follow Supplementary Figure S1.

**
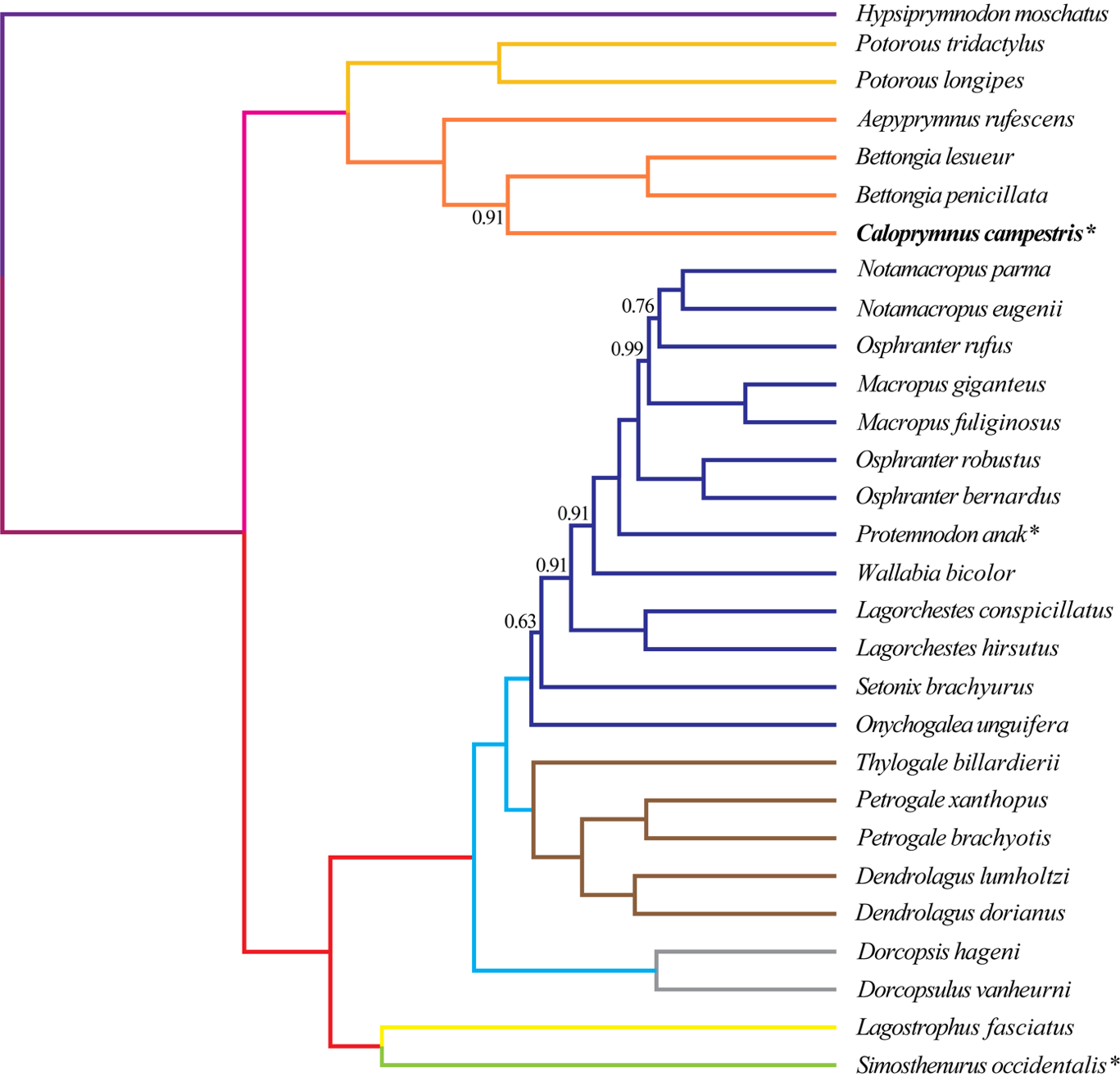
**

**Supplementary Figure S5. Bayesian consensus tree of the partitioned mitogenome dataset generated by *BEAST* 2.2.1^36^.** BPP support values (<1.0) are indicated at relevant nodes. Branch colours denoting major clades follow Supplementary Figure S1.

**
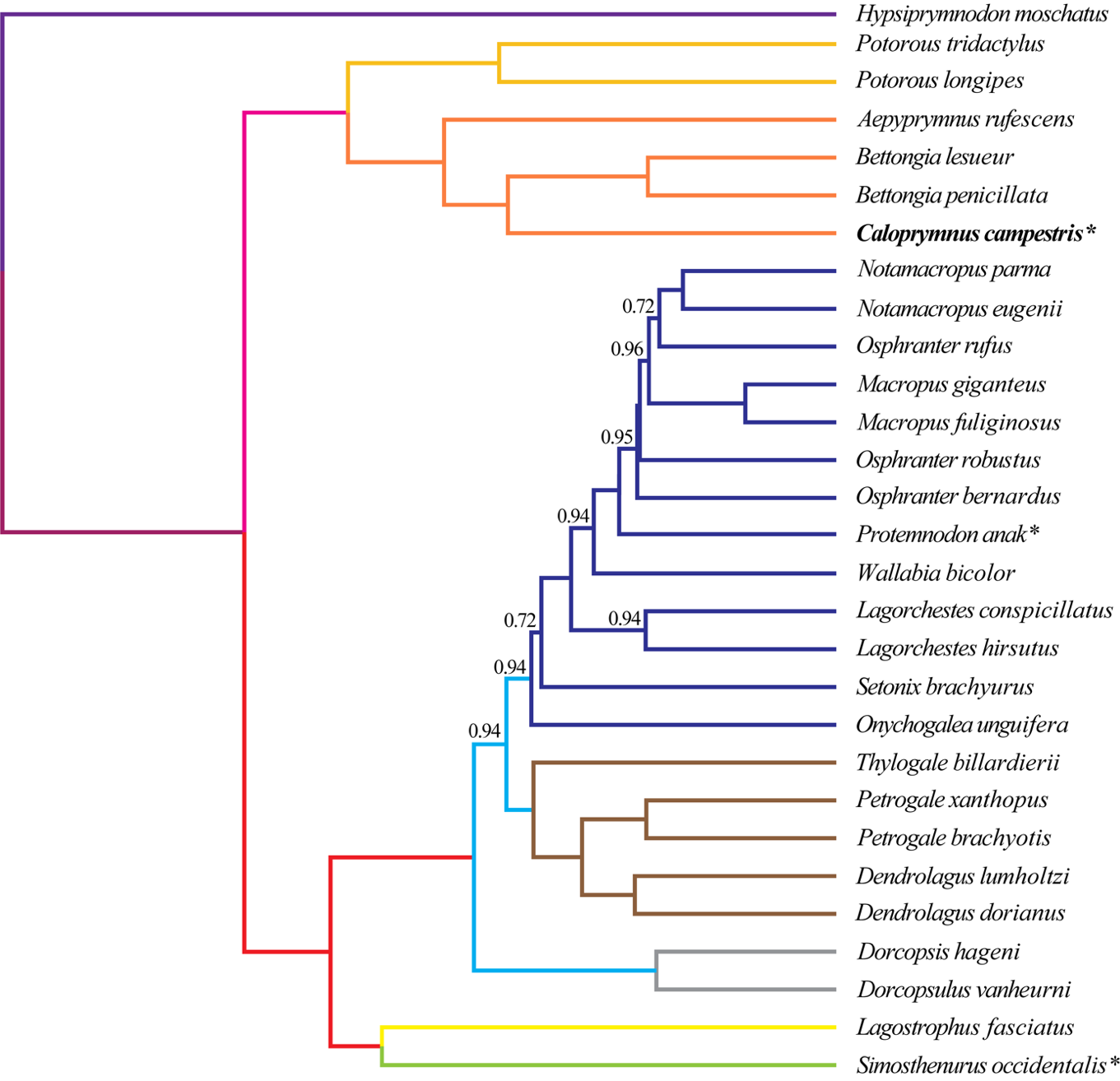
**

**Supplementary Figure S6. Bayesian consensus tree of the non-partitioned mitogenome dataset generated by *BEAST* 2.2.1.** BPP support values (<1.0) are indicated at relevant nodes. Branch colours denoting major clades follow Supplementary Figure S1.

**
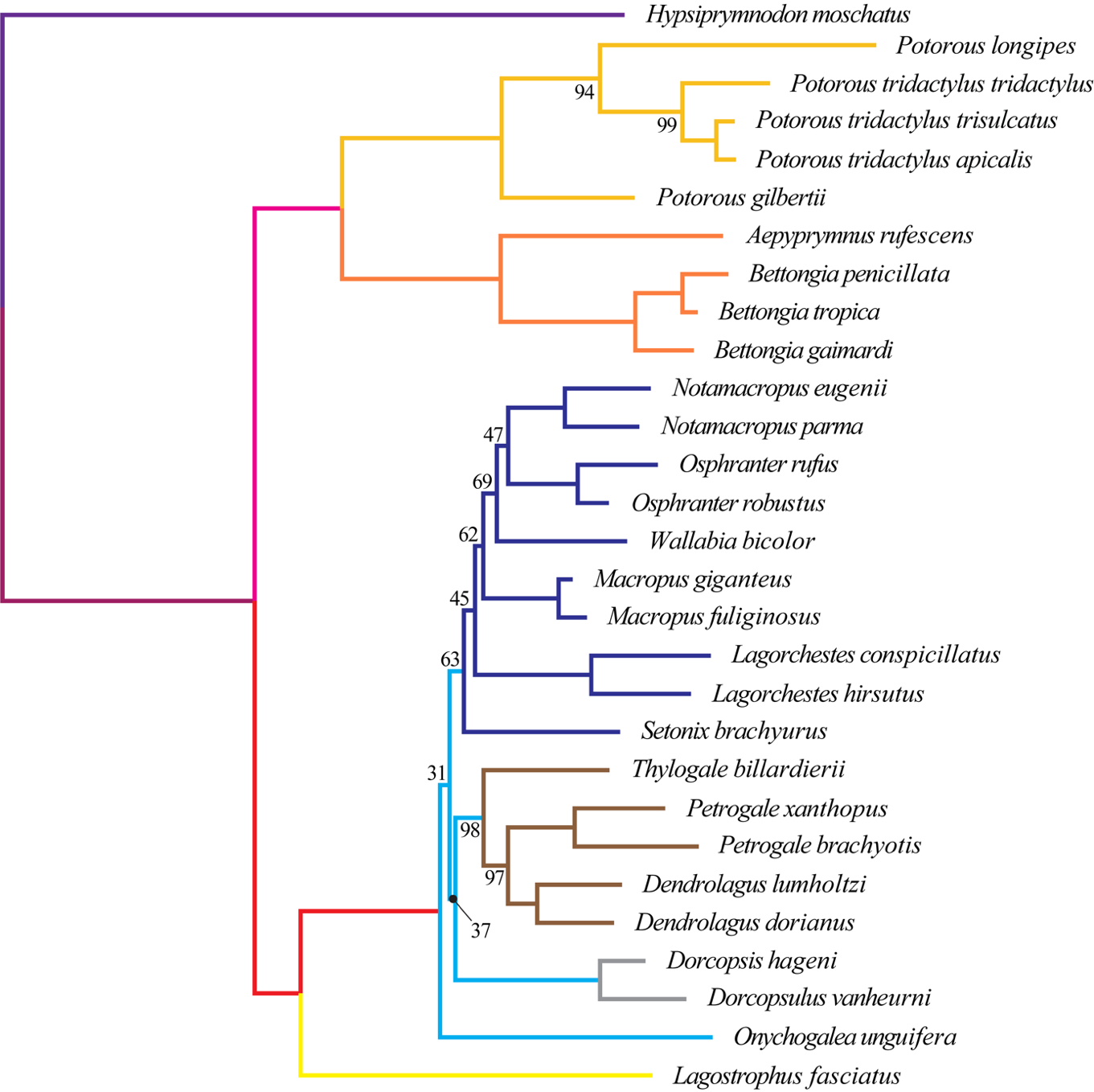
**

**Supplementary Figure S7. Maximum likelihood consensus tree of the nuclear gene sequence (nDNA) dataset generated by *RAxML* 7.2.8.** Bootstrap support values (<100%) are indicated at relevant nodes. Branch colours denoting major clades follow Supplementary Figure S1.

**
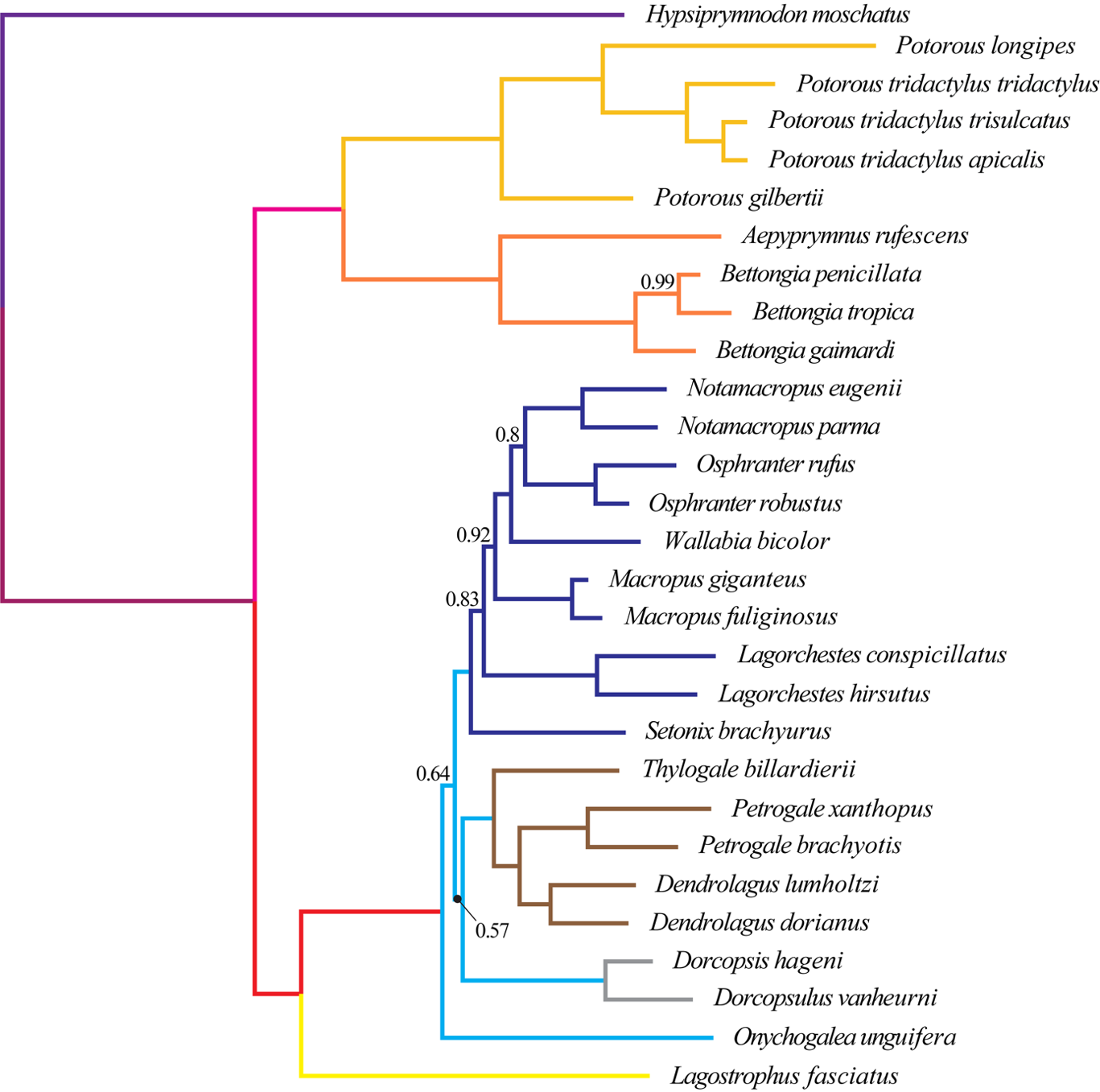
**

**Supplementary Figure S8. Bayesian consensus tree of the nDNA dataset generated by *MrBayes* 3.2.7.** BPP support values (<1.0) are indicated at relevant nodes. Branch colours denoting major clades follow Supplementary Figure S1.

**
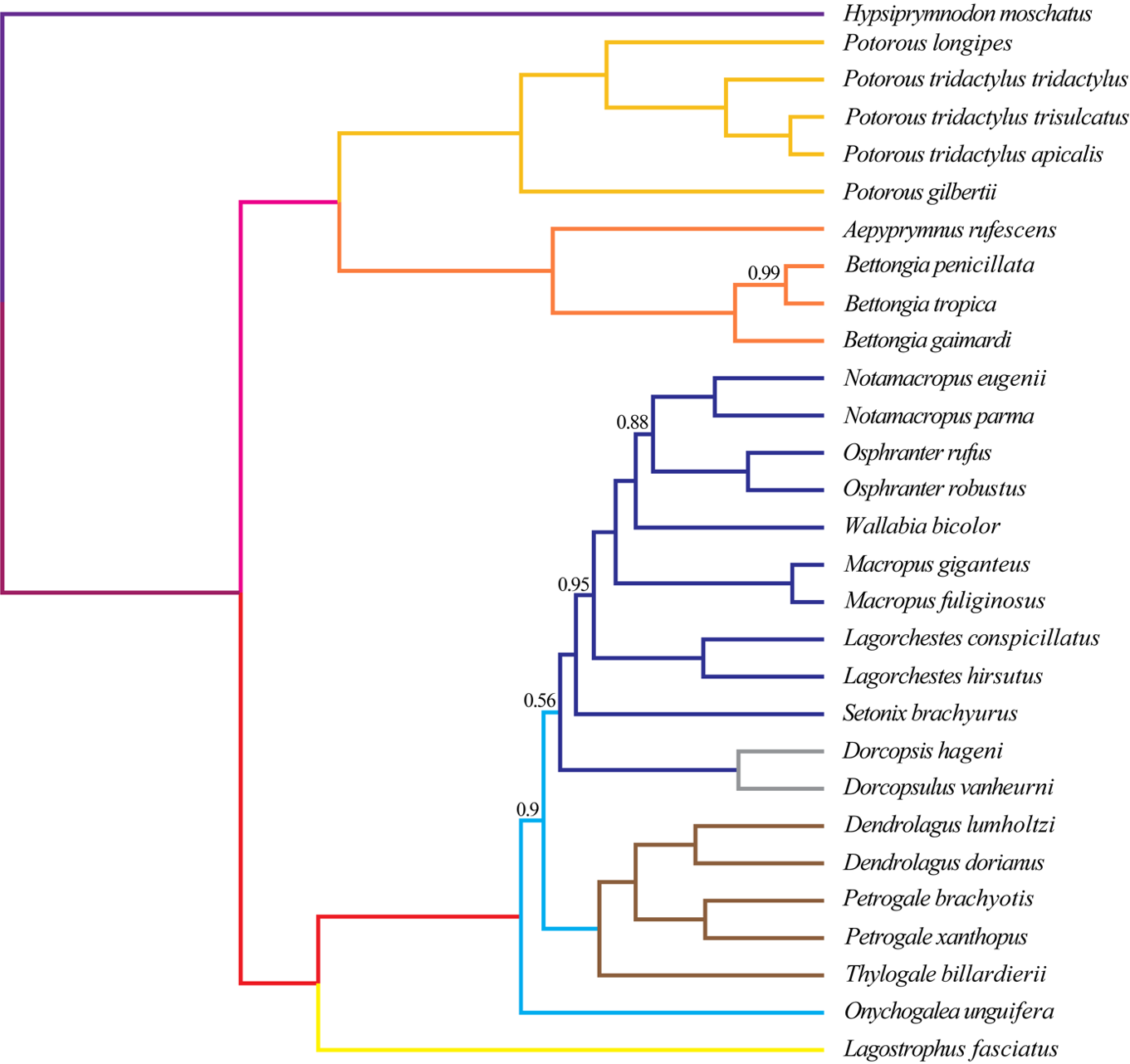
**

**Supplementary Figure S9. Bayesian consensus tree of the nDNA dataset generated by *BEAST* 2.2.1.** BPP support values (<1.0) are indicated at relevant nodes. Branch colours denoting major clades follow Supplementary Figure S1.

**
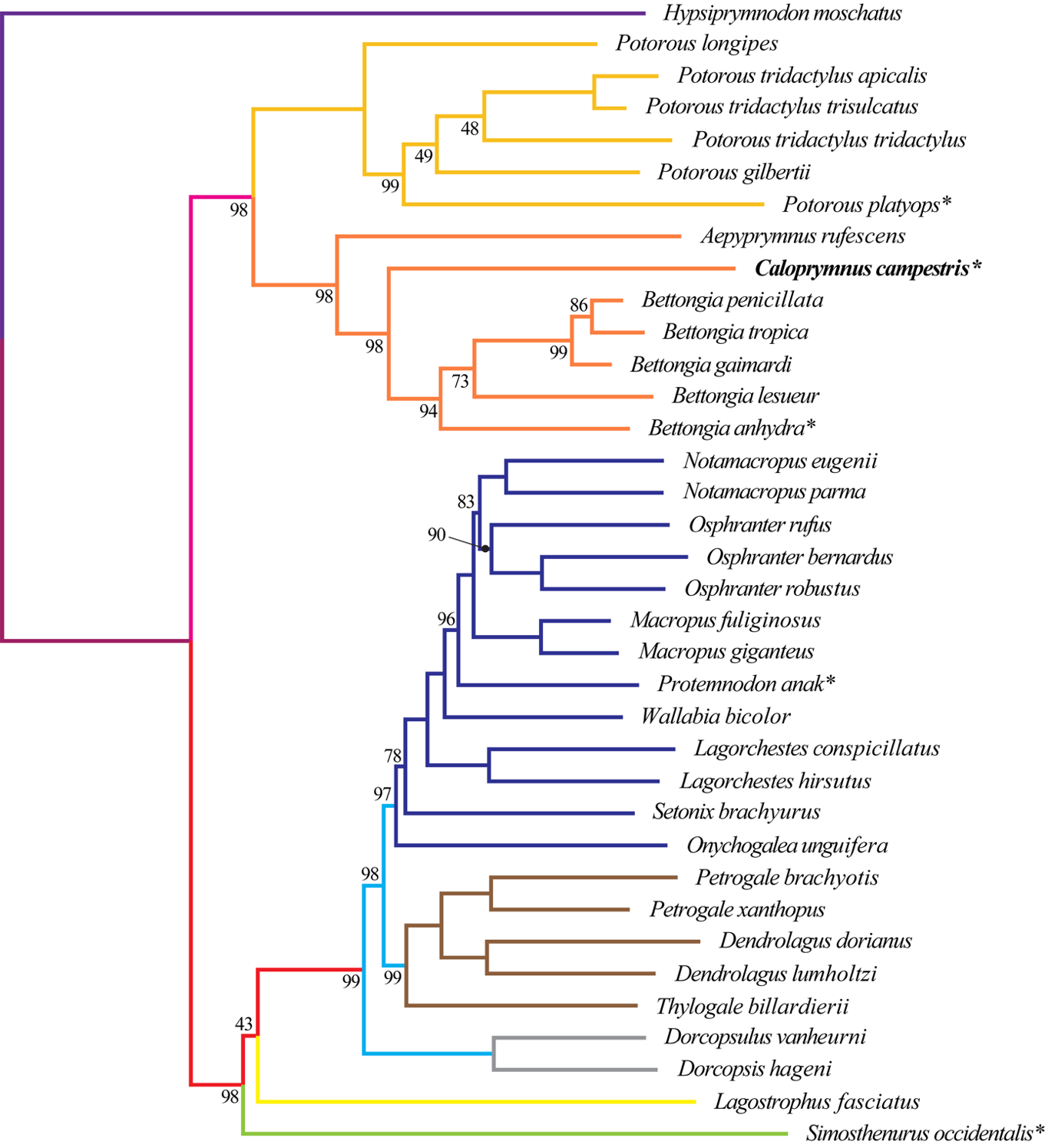
**

**Supplementary Figure S10. Maximum likelihood consensus tree of the non-partitioned mitogenome/mitochondrial gene sequence (mtDNA)/nDNA dataset generated by *RAxML* 7.2.8.** Bootstrap support values (<100%) are indicated at relevant nodes. Branch colours denoting major clades follow Supplementary Figure S1.

**
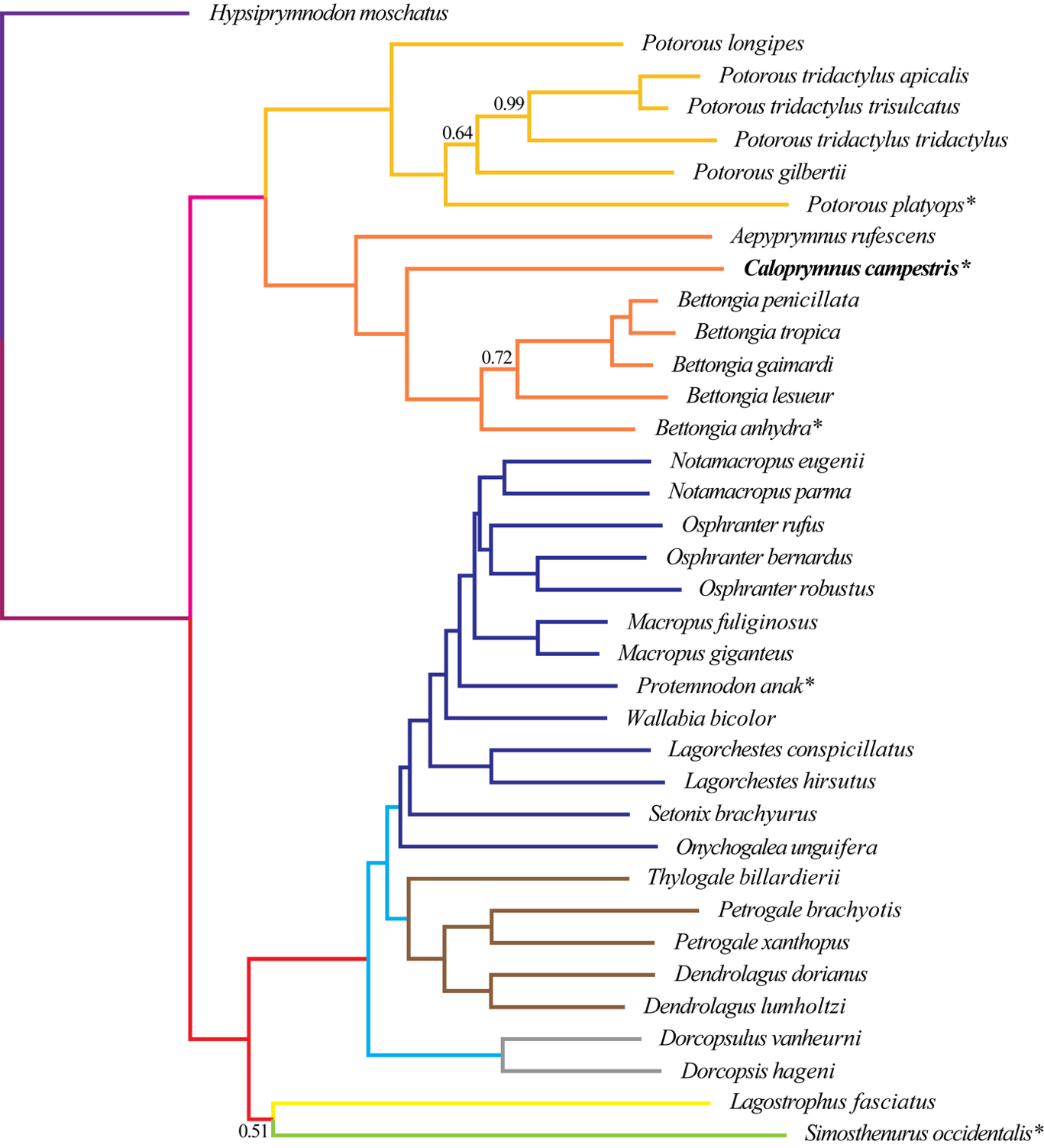
**

**Supplementary Figure S11. Bayesian consensus tree of the non-partitioned mitogenome/mtDNA/nDNA dataset generated by *MrBayes* 3.2.7.** BPP support values (<1.0) are indicated at relevant nodes. Branch colours denoting major clades follow Supplementary Figure S1.

**
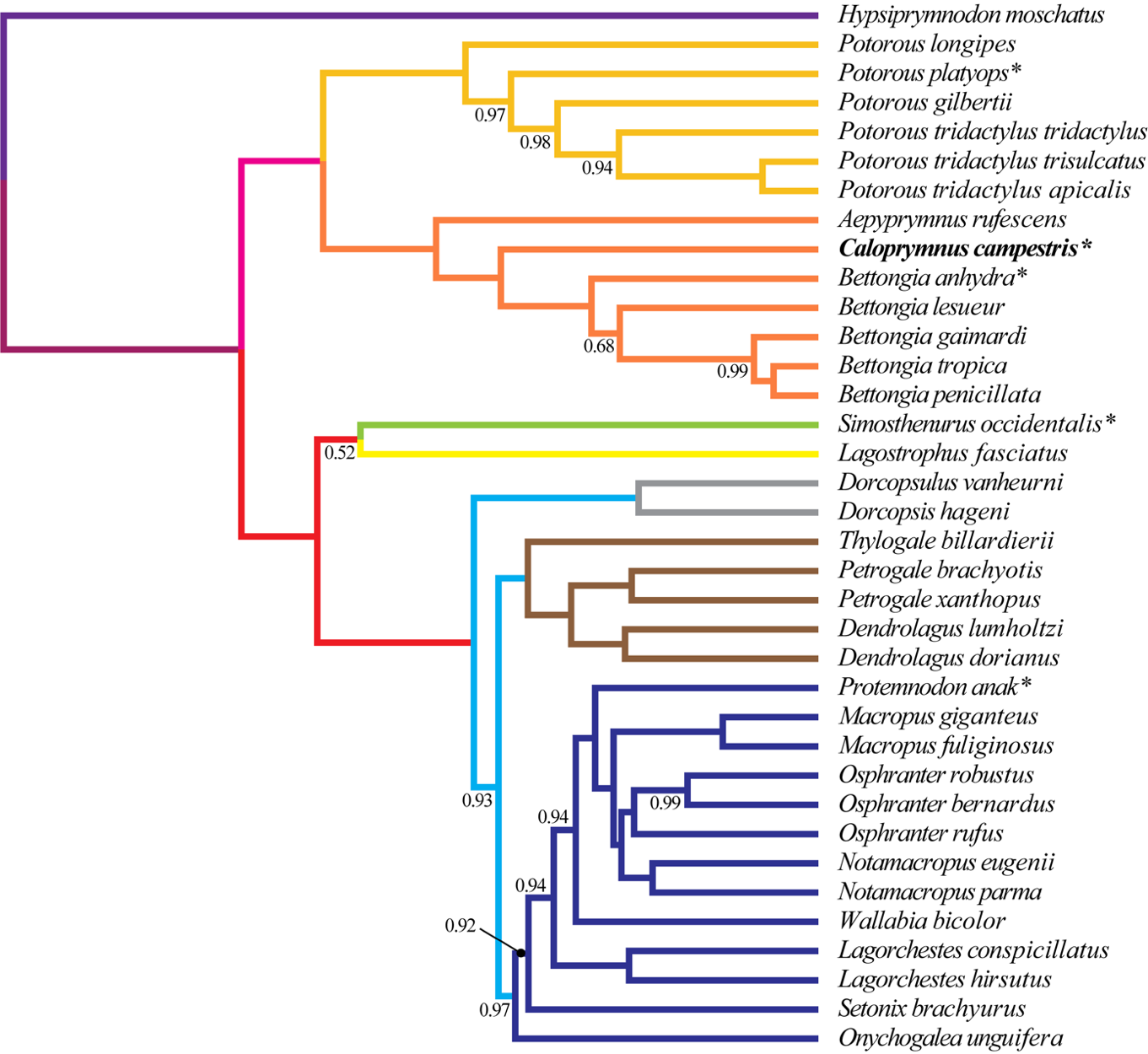
**

**Supplementary Figure S12. Bayesian consensus tree of the non-partitioned mitogenome/mtDNA/nDNA dataset generated by *BEAST* 2.2.1.** BPP support values (<1.0) are indicated at relevant nodes. Branch colours denoting major clades follow Supplementary Figure S1.

**
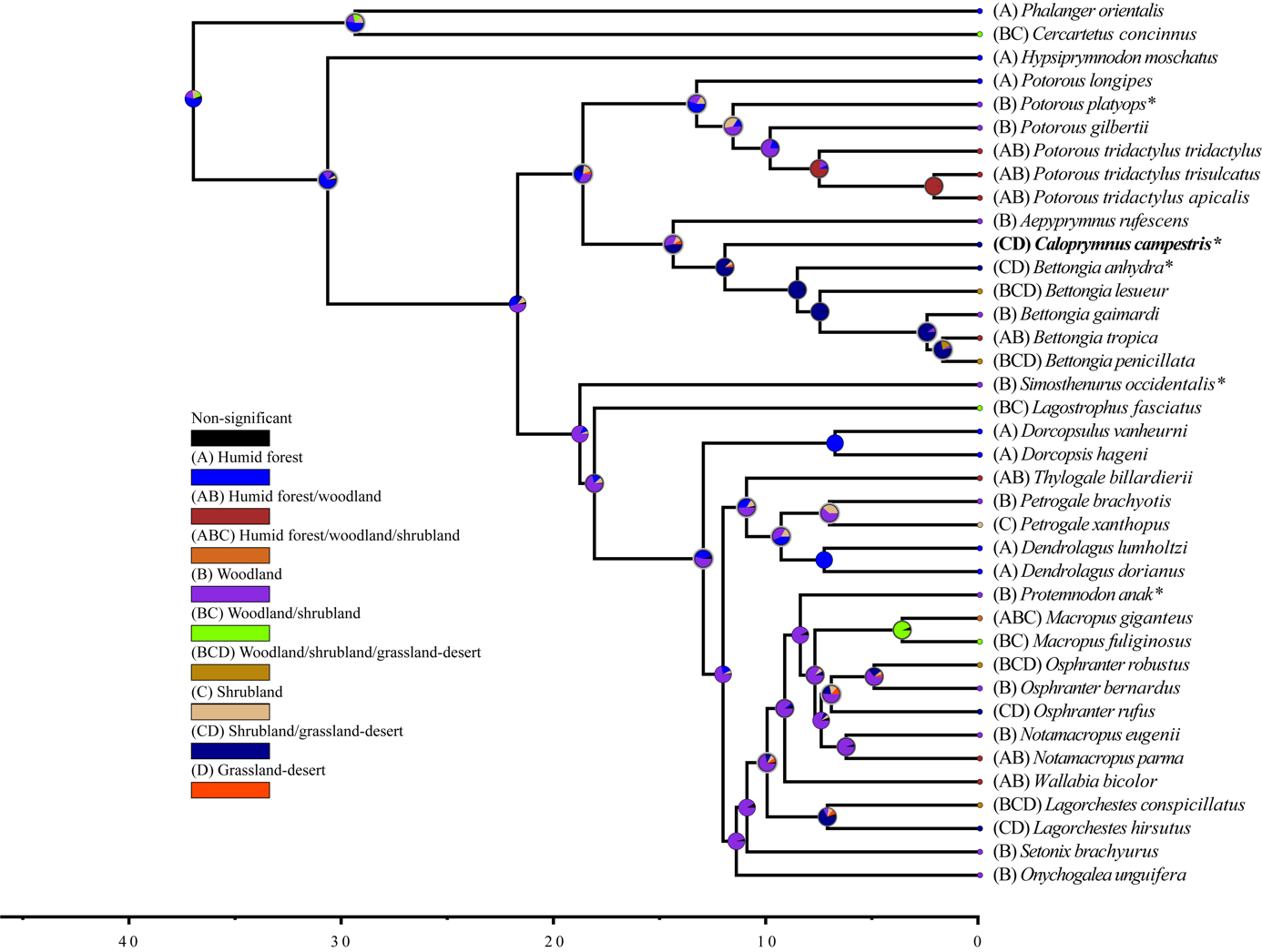
**

**Supplementary Figure S13. Node dated (Ma) consensus tree of the partitioned mitogenome/mtDNA/nDNA dataset generated by *Beast* with ancestral areas derived using the selected BAYAREALIKE+J model from** ***BioGeoBEARS*^37^.** Dispersal (dark grey circles) and vicariance (light grey circles) events are indicated at relevant nodes. Habitat codes are listed for each taxon (right) and explained in the text.

**
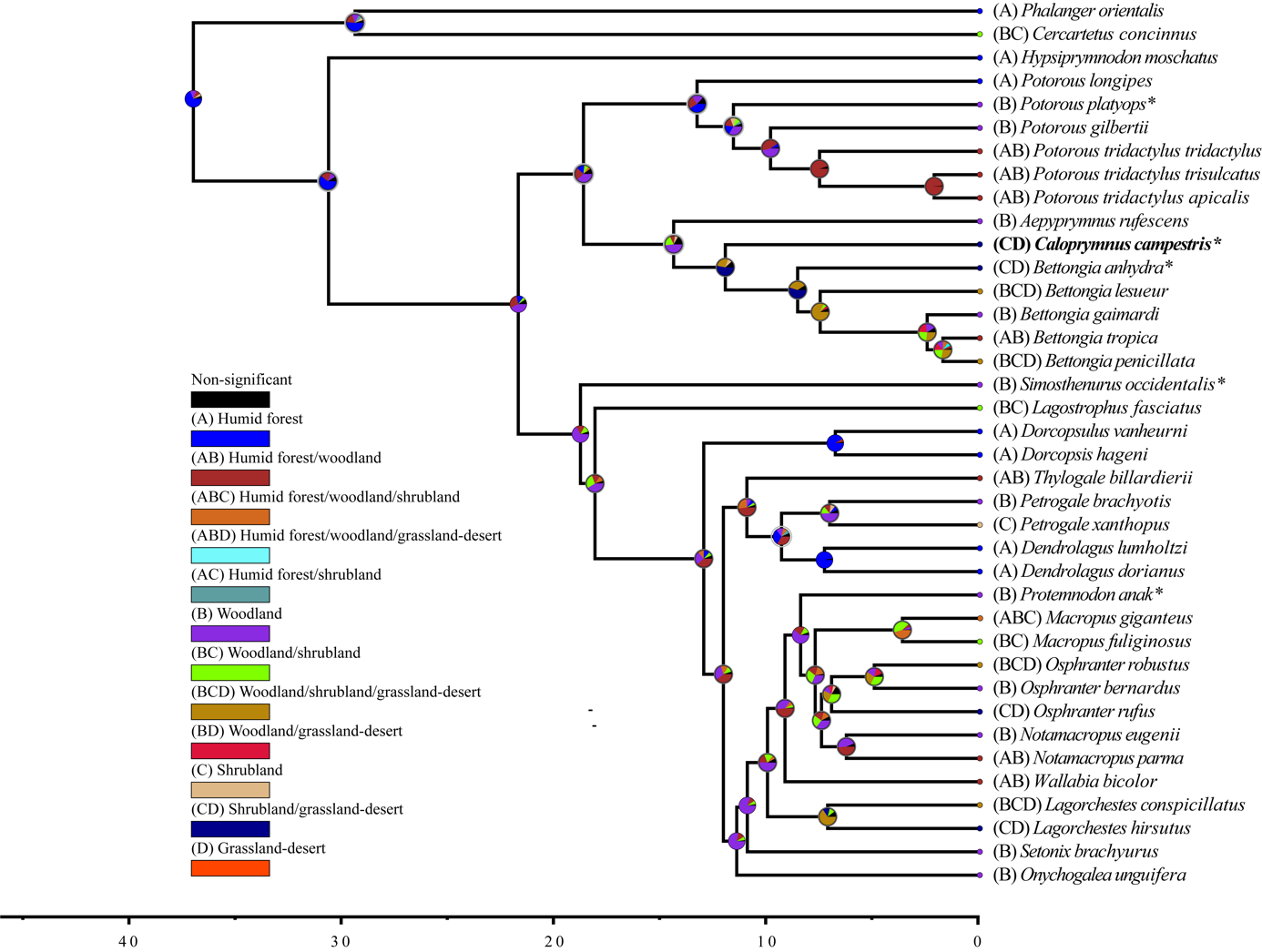
**

**Supplementary Figure S14. Node dated (Ma) consensus tree of the partitioned mitogenome/mtDNA/nDNA dataset generated by *BEAST* with BBM ancestral range reconstructions from *RASP 4*^40^.** Dispersal (dark grey circles) and vicariance (light grey circles) events are indicated at relevant nodes. Habitat codes are listed for each taxon (right) and explained in the text.

**Systematics**

The higher-level systematics of Macropodiformes (the most inclusive clade containing Macropodoidea^14^) has long been contentious^26,120^, with multiple ranking and phylogeny-based classification schemes currently in use^3,14,26,50,120^. To minimise ambiguities in our description of crown macropodoid inter-relationships, we therefore propose new formal phylogenetic definitions for Macropodiformes and all higher-level constituent clades that have received robust analytical support. Our preferred phylogenetic nomenclature (Table 1) builds on the taxonomic schemes of Kear & Cooke^26^, Jackson & Groves^75^, Den Boer & Kear^14^, Eldridge *et al*.^120^ and Beck *et al*.^50^. Our clade-type designations follow the conceptual framework of Lee^121^ where crown node-based definitions are implicated by extant taxa only, as opposed to stem-based definitions which employ both extant and fossil taxa.

**Unranked clade Macropodia, new clade**

Although elsewhere treated as an equivalent to Macropodidae^3,9,64,122^ or Macropodoidea^49^, our concept of Macropodoidea *sensu stricto* (Table 1) as the clade encapsulating the entire crown radiation to the exclusion of demonstrable stem taxa, such as *Palaeopotorous priscus*^14^ and possibly balbarids (“fanged kangaroos”^120^)^26,123–126^, necessitates designation of a new name^50^ for the most inclusive subclade containing Potoroidae + Macropodidae (kangaroos, wallaroos, wallabies, pademelons, tree-kangaroos, bettongs and potoroos) to the exclusion of Hypsiprymnodontidae (rat-kangaroos)^120^. This clade is unanimously resolved by all recent crown macropodoid phylogenies^12,46–49^ and accommodates for fossil taxa that are variously nested along the potoroid and/or macropodid stems^3,9,14,26,50,127–132^.

**Phylogenetic definitions**

All phylogenetic definitions have been officially registered on the *PhyloCode* international clade names repository *RegNum* (<https://www.phyloregnum.org/>).

**Macropodiformes** Kirsch, Springer & Lapointe, 1997 (this study), converted clade name

**Registration number.** 775.

**Phylogenetic definition.** The most inclusive clade including *Balbaroo nalima* *Hypsiprymnodon moschatus*, *Potorous tridactylus* and *Macropus giganteus*, but excluding *Cercartetus concinnus* *and Phalanger orientalis*. Maximum total-clade definition.

**Reference phylogeny.** The phylogenetic hypothesis presented in Fig. 2, together with the fossil-based phylogenetic hypotheses shown in figs S8B and S9B of Den Boer & Kear^14^ and fig. 10 of Butler *et al*.^126^.

**Composition.** The composition is based on the reference phylogenies to incorporate Balbaridae (see ‘Balbaridae’), Hypsiprymnodontidae (see ‘Hypsiprymnodontidae’) and Macropodia (see ‘Macropodia’). *Palaeopotorous priscus* is also a stem macropodiform^14^.

**Macropodoidea** Gray, 1821 (this study), converted clade name

**Registration number.** 776.

**Phylogenetic definition.** The least inclusive clade including *Hypsiprymnodon moschatus*, *Potorous tridactylus* and *Macropus giganteus*. Minimum crown clade definition.

**Reference phylogeny.** The phylogenetic hypothesis presented in Fig. 2.

**Composition.** The composition is based on the reference phylogeny to incorporate Hypsiprymnodontidae (see ‘Hypsiprymnodontidae’) and Macropodia (see ‘Macropodia’).

**Balbaridae** Kear & Cooke, 2001 (this study), converted clade name

**Registration number.** 777.

**Phylogenetic definition.** The most inclusive clade including *Balbaroo nalima*, but excluding *Hypsiprymnodon moschatus*, *Potorous tridactylus* and *Macropus giganteus*. Maximum clade definition.

**Reference phylogeny.** The phylogenetic hypotheses presented in figs S8B and S9B of Den Boer & Kear^14^ and fig. 10 in Butler *et al*.^126^.

**Composition.** The composition is based on the reference phylogenies to incorporate the species of *Nambaroo*^26,126^, *Ganawamaya*^126^ and *Balbaroo*^125^ with *Wururoo dayamayi*^26,125^.

**Hypsiprymnodontidae** Collett, 1887 (this study), converted clade name

**Registration number.** 778.

**Phylogenetic definition.** The most inclusive clade including *Hypsiprymnodon moschatus* and *Propleopus oscillans*, but excluding *Balbaroo nalima*, *Potorous tridactylus* and *Macropus giganteus*. Maximum clade definition.

**Reference phylogeny.** The phylogenetic hypothesis presented in Fig. 2, together with the fossil-based phylogenetic hypothesis shown in fig. S8 of Den Boer & Kear^14^.

**Composition.** The composition is based on the reference phylogenies to incorporate Hypsiprymnodontinae (see ‘Hypsiprymnodontinae’) and Propleopinae (see ‘Propleopinae’).

**Hypsiprymnodontinae** Collett, 1887 (this study), converted clade name

**Registration number.** 779.

**Phylogenetic definition.** The most inclusive clade including *Hypsiprymnodon moschatus*, but excluding *Propleopus oscillans*. Maximum clade definition.

**Reference phylogeny.** The phylogenetic hypothesis presented in Fig. 2, together with the fossil-based phylogenetic hypothesis shown in fig. S8 of Den Boer & Kear^14^.

**Composition.** The composition is based on the reference phylogenies to incorporate *Hypsiprymnodon moschatus* with other fossil species assigned to the genus *Hypsiprymnodon*^133^.

**Propleopinae** Archer & Flannery, 1985 (this study), converted clade name

**Registration number.** 780.

**Phylogenetic definition.** The most inclusive clade including *Propleopus oscillans*, but excluding *Hypsiprymnodon moschatus*. Maximum clade definition.

**Reference phylogeny.** The phylogenetic hypothesis presented in fig. S8 of Den Boer & Kear^14^.

**Composition.** The composition is based on the reference phylogeny to incorporate the species of *Propleopus* and *Ekaltadeta* with *Jackmahoneya toxoniensis*^14,26^.

**Macropodia** new clade name

**Registration number.** 781.

**Phylogenetic definition.** The least inclusive clade including *Potorous tridactylus* and *Macropus giganteus*, but excluding *Hypsiprymnodon moschatus*. Minimum crown clade definition.

**Reference phylogeny.** The phylogenetic hypothesis presented in Fig. 2.

**Composition.** The composition is based on the reference phylogeny to incorporate Potoroidae (see ‘Potoroidae’) and Macropodidae (see ‘Macropodidae’).

**Potoroidae** Pearson, 1950 (this study), converted clade name

**Registration number.** 782.

**Phylogenetic definition.** The least inclusive clade including *Potorous tridactylus* and *Aepyprymnus rufescens*, but excluding *Hypsiprymnodon moschatus* and *Macropus giganteus*. Minimum crown clade definition.

**Reference phylogeny.** The phylogenetic hypothesis presented in Fig. 2.

**Composition.** The composition is based on the reference phylogeny to incorporate Potoroinae (see ‘Potoroinae’) and Bettonginae (see ‘Bettonginae’).

**Potoroinae** Gray, 1821 (this study), converted clade name

**Registration number.** 783.

**Phylogenetic definition.** The least inclusive clade including *Potorous tridactylus*, but excluding *Aepyprymnus rufescens*. Minimum crown clade definition.

**Reference phylogeny.** The phylogenetic hypothesis presented in Fig. 2.

**Composition.** The composition is based on the reference phylogeny to incorporate the species of *Potorous*.

**Bettonginae** Bensley, 1903 (this study), converted clade name

**Registration number.** 784.

**Phylogenetic definition.** The least inclusive clade including *Aepyprymnus rufescens*, but excluding *Potorous tridactylus*. Minimum crown clade definition.

**Reference phylogeny.** The phylogenetic hypothesis presented in Fig. 2.

**Composition.** The composition is based on the reference phylogeny to incorporate the species of *Bettongia* with *Aepyprymnus rufescens* and *Caloprymnus campestris*.

**Macropodidae** Gray, 1821 (this study), converted clade name

**Registration number.** 785.

**Phylogenetic definition.** The most inclusive clade including *Simosthenurus occidentalis*, *Lagostrophus fasciatus* and *Macropus giganteus*, but excluding *Potorous tridactylus* and *Hypsiprymnodon moschatus*. Maximum clade definition.

**Reference phylogeny.** The phylogenetic hypothesis presented in Fig. 2.

**Composition.** The composition is based on the reference phylogeny to incorporate Sthenurinae (see ‘Sthenurinae’), Lagostrophinae (see ‘Lagostrophinae’) and Macropodinae (see ‘Macropodinae’).

**Sthenurinae** Glauert, 1926 (this study), converted clade name

**Registration number.** 786.

**Phylogenetic definition.** The most inclusive clade including most inclusive clade including *Simosthenurus occidentalis*, but excluding *Lagostrophus fasciatus* and *Macropus giganteus*. Maximum clade definition.

**Reference phylogeny.** The phylogenetic hypothesis presented in Fig. 2, together with the fossil-based phylogenetic hypotheses shown in figs S8B and S9B of Den Boer & Kear^14^, fig. 21 of Prideaux^134^ and fig. 18 of Black *et al*.^125^.

**Composition.** The composition is based on the reference phylogenies to incorporate the species of *Sthenurus*, *Archaeosimos*, *Simosthenurus* and *Procoptodon*^134^ with *Hadronomas puckridgi*^14,26^, *Rhizosthenurus flanneryi*^125,135^ and *Wanburoo hilarus*^3,14^. The species of *Wabularoo* might also represent basally divergent sthenurines^14,130^.

**Lagostrophinae** Prideaux & Warburton, 2010 (this study), converted clade name

**Registration number.** 787.

**Phylogenetic definition.** The most inclusive clade including *Lagostrophus fasciatus*, but excluding *Simosthenurus occidentalis* and *Macropus giganteus*. Maximum clade definition.

**Reference phylogeny.** The phylogenetic hypothesis presented in Fig. 2, together with the fossil-based phylogenetic hypothesis shown in figs S8B and S9B of Den Boer & Kear^14^.

**Composition.** The composition is based on the reference phylogeny to incorporate *Lagostrophus fasciatus* with *Tjukuru wellsi*^14,136^ and the species of *Troposodon*^137^.

**Macropodinae** Gray, 1821 (this study), converted clade name

**Registration number.** 788.

**Phylogenetic definition.** The most inclusive clade including *Macropus giganteus*, but excluding *Simosthenurus occidentalis* and *Lagostrophus fasciatus*. Maximum clade definition.

**Reference phylogeny.** The phylogenetic hypothesis presented in Fig. 2.

**Composition.** The composition is based on the reference phylogeny to incorporate Dorcopsini (see ‘Dorcopsini’), Dendrolagini (see ‘Dendrolagini’) and Macropodini (see ‘Macropodini’).

**Dorcopsini** Prideaux & Warburton, 2010 (this study), converted clade name

**Registration number.** 789.

**Phylogenetic definition.** The least inclusive clade including *Dorcopsis hageni*, but excluding *Dendrolagus lumholtzi* and *Macropus giganteus*. Minimum crown clade definition.

**Reference phylogeny.** The phylogenetic hypothesis presented in Fig. 2.

**Composition.** The composition is based on the reference phylogeny to incorporate the species of *Dorcopsis* and *Dorcopsulus*^87^. *Dorcopsoides fossilis* might also represent a basally divergent dorcopsin^3^.

**Dendrolagini** Flannery, 1989 (this study), converted clade name

**Registration number.** 790.

**Phylogenetic definition.** The least inclusive clade including *Dendrolagus lumholtzi*, but excluding *Dorcopsis hageni* and *Macropus giganteus*. Minimum crown clade definition.

**Reference phylogeny.** The phylogenetic hypothesis presented in Fig. 2.

**Composition.** The composition is based on the reference phylogeny to incorporate the species of *Dendrolagus*^73.98^, *Thylogale*^72^ and *Petrogale*^138^. The extinct species of *Bohra* are also the immediate sister lineage of *Dendrolagus*^3^.

**Macropodini** Flannery, 1989 (this study), converted clade name

**Registration number.** 791.

**Phylogenetic definition.** The least inclusive clade including *Macropus giganteus*, but excluding *Dorcopsis hageni* and *Dendrolagus lumholtzi*. Minimum crown clade definition.

**Reference phylogeny.** The phylogenetic hypothesis presented in Fig. 2.

**Composition.** The composition is based on the reference phylogeny to incorporate the species of *Macropus*, *Osphranter*, *Notamacropus*^12^, *Lagorchestes* and *Onychogalea* with *Wallabia bicolor* and *Setonix brachyurus*. The extinct species of *Protemnodon*^139^, *Kurrabi*^140^ and *Congruus*^141^ with *Prionotemnus palankarinnicus* and *Baringa nelsonensis* are also recovered within Macropodini^3^.

**Node dating calibrations**

**Macropodiformes versus Phalangeriformes split (root node)**

Minimimum bound. 27.82 Ma.

Maximum bound. 54.65 Ma.

Explanation. Woodburne *et al*.^142^ documented the historically oldest identified macropodiform and phalangeriform fossils, including the informally named “geologically oldest potoroine kangaroo yet known”, “*Kyeema mahoneyi*”, from the “Winyardiid” interval representing Zone A of the Etadunna Formation in South Australia. Woodburne *et al*.^142^ constrained the entire Etadunna Formation sequence to between 25.7–24.2 Ma based on magnetostratigraphic data; however, Metzger & Retallack^57^ revised this to between 26.01–23.6 Ma. Megirian *et al*.^143^ otherwise specifically dated their Etadunna Formation Zone A at 25.2–24.9 Ma following the magnetic polarity chrons assigned by Woodburne *et al*.^142^. Murray & Megirian^144^ additionally reported isolated teeth attributable to potoroids and possible phalangerids from the upper Oligocene Pwerte Marnte Marnte Local Fauna (LF) in the Northern Territory, which Megirian *et al*.^143^ considered to be older than Etadunna Formation Zone A, and thus part of their ‘pre-Etadunnan’ Australian Land Mammal Age (LMA) series bounded at >30 Ma. Archer *et al*.^145^ also listed numerous unequivocal macropodiform and phalangeriform taxa from uppermost Oligocene Zone A deposits of the Riversleigh World Heritage Area in Queensland (see Arena *et al*.^146^ for a detailed discussion of the Riversleigh zonation scheme). These included the stratigraphically wide-ranging burramyid, *Burramys brutyi*, which Archer *et al*.^147^ delimited with a maximum age of 25 Ma. However, García-Navas *et al*.^13^ recalibrated the oldest estimated age of the Riversleigh Faunal Zone A assemblages from the base of the Chattian after Woodhead *et al*.^148^, which equates to 27.82 Ma from Cohen *et al*.^149^. We therefore employ this minimum bound for our constrained divergence of Macropodiformes and Phalangeriformes.

Our maximum bound follows Meredith *et al*.^150^, who used the radiometric date of 54.6±0.05 Ma from the lower Eocene Tingamarra LF of Murgon in Queensland. This assemblage incorporates the geologically oldest unequivocal australidelphian marsupial fossils^151^, and accommodates for all previous estimates of the earliest split between Macropodiformes and Phalangeriformes within Diprotodontia^5,50,152,153^.

***Cercartetus* versus *Phalanger* split (crown Phalangeroidea)**

Minimimum bound. 27.82 Ma.

Maximum bound. 54.65 Ma.

Explanation. The oldest fossil occurrences of *Cercartetus* are currently restricted to lower Pliocene cave deposits^140,154^. However, Brammall & Archer^155^ alternatively summarised the much more extensive stratigraphical distribution of its sister taxon *Burramys*, whose oldest fossils include *Burramys wakefieldi*^156^, from the uppermost Oligocene Ngama LF representing Etadunna Formation Zone D^142^, and *Burramys brutyi*^157^ from the Riversleigh Zone A White Hunter Site^145.146^. Archer *et al*.^147^ mentioned that the upper Oligocene sequences at Riversleigh might be slightly younger than those in South Australia, but designated a consistent maximum age for both at 25 Ma. Megirian *et al*.^143^ otherwise specifically constrained their Etadunna Formation Zone D to 24.1–24 Ma, which postdates the maximum collective limit for the Riversleigh Zone A sites at 27.82 Ma^13^.

Similarly, the genus-level affinities of many fossil phalangerids (including *Phalanger*^158^) are ambiguous, although the most diagnostic specimens occur at localities correlated with the lower Miocene Riversleigh Zone B^159^. However, Case *et al*.^160^ described *Eocuscus sarastamppi* from the Ditjimanka LF, which correlates with the upper Oligocene Etadunna Formation Zone B at “ca. 25 [Ma]”, or 24.9–24.6 Ma after Megirian *et al*.^143^. Given that Murray & Megirian^144^ also identified potential phalangerids from the ‘pre-Etadunnan’ Pwerte Marnte Marnte LF at >30^143^, we consider the most stable minimum bound to be 27.82 Ma because this accommodates for these fossils relative to the oldest estimated age of Riversleigh Faunal Zone A^13^. Our maximum bound alternatively follows Meredith *et al*.^152^, who cited the radiometric date of 54.6±0.05 Ma from the lower Eocene Tingamarra LF to most feasibly incorporate the base of Phalangeroidea.

**Hypsiprymnodontidae versus Potoroidae + Macropodidae split (crown Macropodoidea)**

Minimimum bound. 15.97 Ma.

Maximum bound. 27.82 Ma.

Explanation. Den Boer & Kear^14^ established *Palaeopotorous priscus* from the uppermost Oligocene Tarkarooloo LF of the Namba Formation in South Australia as the oldest identifiable stem macropodiform. Woodburne *et al*.^142^ correlated the Tarkarooloo LF with Zone D of the laterally equivalent Etadunna Formation. Megirian *et al*.^143^ specifically constrained Etadunna Formation Zone D to 24.1–24 Ma. Notably, though, the basally branching macropodoids *Ngamaroo archeri* and *Purtia mosaicus* occur within Etadunna Formation Zone D and Zone C, respectively^122,127^. Following the chronostratigraphic succession of Megirian *et al*.^143^, this would suggest a maximum bound of up to 24.6 Ma. However, the phylogenetic relationships of *N. archeri* and *P. mosaicus* are both unresolved^125–132^, which is problematic for node dating purposes^48^. Moreover, various putative stem potoroids and macropodids have been described from deposits correlated with the upper Oligocene Riversleigh Zone A^127–132^. All of these taxa are phylogenetically ambiguous leading to uncertainty over their classifications^120^. As a result, we conservatively set our maximum stratigraphic bound for the root split within crown Macropodoidea (= Hypsiprymnodonidae versus Potoroidae + Macropodidae) at 27.82 Ma to accommodate for the recalibrated age of Riversleigh Faunal Zone A after García-Navas *et al*.^13^.

The minimum constraint for divergence of Hypsiprymnodontidae (as defined by the crown genus *Hypsiprymnodon*^14,50^) versus Macropodia is delimited by the stratigraphically earliest occurrence of *Hypsiprymnodon*, which has been identified from a range of fossil sites spanning the entire Riversleigh Faunal Zone B^133^; this equates to the lower Miocene B1–B3 intervals of Arena *et al*.^146^. Amongst these, Neville’s Garden Site and Camel Sputum Site were both radiometrically dated by Woodhead *et al*.^148^ at 18.24 ± 0.29 and 17.75 ± 0.78, respectively. Nonetheless, Woodhead *et al*.^148^ also cited a biocorrelated early Miocene age range of 23–16 Ma for Riversleigh Faunal Zone B, which we recalibrate to 23.03–15.97 Ma using the base of the Aquitanian to top of the Burdigalian after Cohen *et al*.^149^. Our minimum bound for the root divergence within crown Macropodoidea is thus set at 15.97 Ma, which additionally accommodates for many of the youngest mean estimates generated by Brennan^48^, Cascini *et al*.^49^ and Beck *et al*.^50^ using their tip-dating and total evidence dating approaches.

**Potoroidae versus Macropodidae split (crown Macropodia)**

Minimimum bound. Not designated.

Maximum bound. Not designated.

Explanation. Meredith *et al*.^46^ fixed their maximum limit for the split of Potoroidae versus Macropodidae at 24.8 Ma using a stratigraphic bound defined by the absence of demonstrably assignable crown group fossils in Zone A of the Etadunna Formation^142^. This calibration approximates the 24.7 Ma prior employed by Cascini *et al*.^49^. The oldest fossils currently assigned to Potoroidae and/or Macropodidae derive from Riversleigh Zone A^128–132,145^, which has been stratigraphically delimited at 27.82 Ma^13^. These include the putative stem potoroid *Gumardee* spp.^132,161^, which occurs together with indeterminate “potoroid” fossils in the upper Oligocene Tarkarooloo LF of the Namba Formation^162^. Megirian *et al*.^143^ considered the Tarkarooloo LF assemblage ‘Pre-Etadunnan’, and thus assigned an approximate boundary estimate of >30 Ma. Problematically, however, the various species of *Gumardee* are not recovered as stem potoroids in other phylogenies^126,131,133^, and isolated “potoroid” teeth from the Tarkarooloo LF^162^ have alternatively been interpreted as stem macropodiforms^14^. Similarly, the informally defined “potoroine”, “*Kyeema mahoneyi*”, from Etadunna Formation Zone A^142^ (if valid) is designated Macropodiformes *incertae sedis*^26^. The historically posited stem potoroids *Purtia mosaicus*^122^ from the Ngapakaldi LF of Etadunna Formation Zone C (24.6–24.1 Ma^143^), and *Wakiewakie lawsoni*^54,163^ from the Kutjamarpu LF of the Wipajiri Formation in South Australia (= Wipajirian LMA at up to 17.6 Ma^143^) are also phylogenetically unstable^125–132^, and have otherwise been referred to the paraphyletic stem macropodid (contra Flannery *et al*.^161^) grouping “Bulungamayinae”^26,50,123,127^. The next oldest stem potoroid, ‘*Bettongia*’ *moyesi*, from the middle Miocene Riversleigh System C Two Trees Site is phylogenetically equivocal^125–132^ with some studies even suggesting non-potoroid affinities^26,123^. Furthermore, the age of Two Trees Site has been disputed^164^, yet the most recent assessment^146^ has reaffirmed its biocorrelation with Riversleigh System C.

The oldest consistently nested crown macropodid fossil^3,9,12,14,26,48,50,53,123,124–132,135,136,165^ is the basally branching macropodine, *Dorcopsoides fossilis*, from the upper Miocene Alcoota LF of the Waite Formation in the Northern Territory^166^. Megirian *et al*.^143^ radiometrically correlated the Alcoota LF with the Waitean LMA at >5.84 Ma, although competing age estimates have ranged from 7–8 Ma to as much as 12 Ma^9,167^. In addition, *Dorcopsoides fossilis* is coeval with the basally branching sthenurine *Hadronomas puckridgi*^166,168^, and another uncontested basally branching sthenurine, *Rhizosthenurus flanneryi*, has been recovered from the lower-upper Miocene Riversleigh Zone D Encore Site^135^. Encore Site was estimated at around 12 Ma by Megirian *et al*.^143^ and Couzens & Prideaux^9^, although Woodhead *et al*.^148^ listed a range of 11.6–5.3 Ma, or 11.63–5.333 Ma using the base of the Tortonian to top of the Messinian after Cohen *et al*.^149^. Both Prideaux & Warburton^3^ and Couzens & Prideaux^9^ phylogenetically bracketed Sthenurinae within crown Macropodidae, as delimited by the basal-most clade incorporating the extant lagostrophine, *Lagostrophus fasciatus*. They also recovered the possible stem macropodid *Wanburoo hilarus* as a sthenurine, although this taxon has been identified from middle Miocene Riversleigh Zone C sites, including Dome Site^169^, which Arena *et al*.^146^ assigned to the lowermost Riversleigh Zone C1 interval. Woodhead *et al*.^148^ considered Riversleigh Zone C to be between 16–11.6 Ma (with a specific median age of 14.5 Ma allocated for Dome Site by Couzens & Prideaux^9^), or 15.97–11.63 Ma using the base of the Langhian to top of the Serravallian after Cohen *et al*.^149^. Furthermore, Prideaux & Warburton^3^ and Couzens & Prideaux^9^ included another stem macropodid, *Ganguroo bilamina*, within the paraphyletic basal branches of crown Macropodidae. Prideaux & Warburton^3^ derived their original scores for this taxon from undisclosed specimens in the literature, but *G. bilamina* has since been treated as a hypodigm, and split into three species^128,129^ that collectively span the Riversleigh zones B–D — an extraordinarily extended age range of 23.03–5.333 Ma. Critically, Brennan^48^ found that phylogenetic ambiguity surrounding the placement of *G. bilamina* led to inflated divergence estimates for the Potoroidae-Macropodidae split. Consequently, given the difficulty in pinpointing an alternative more stable age prior, we have elected to leave this node undesignated pending the discovery of more definitively attributable crown potoroid and macropodid fossils.

**Potoroinae versus Bettonginae split (crown Potoroidae)**

Minimum bound. 3.6 Ma.

Maximum bound. 15.97 Ma.

Explanation. The oldest possible crown potoroine fossils are referred to *Potorous* sp. from the upper Miocene or lower Pliocene Curramulka LF in the Yorke Peninsula of South Australia^170,171^. Megirian *et al*.^143^ attributed the Curramulka LF to the Tirarian LMA without detailed explanation, but this provides a geochronometric minimum bound of 3.6 Ma, which equates to the top of the Zanclean after Cohen *et al*.^149^. While the corresponding maximum bound is speculative, we use the base of the Langhian^149^ because this is consistent with age priors employed elsewhere^46^.

***Lagostrophus* versus *Simosthenurus* split**

Minimum bound. 4.41 Ma.

Maximum bound. 15.97 Ma.

Explanation. The oldest fossils attributed to *Lagostrophus* are late Pliocene to early Pleistocene in age^172^, with other possible lagostrophines including the species of *Troposodon* and *Tjukuru wellsi* ranging from the Waitean (>5.84 Ma) to Tirarian LMA^136^, with the oldest radiometric date derived from the lower Pliocene Hamilton LF of Victoria^143^. Megirian *et al*.^143^ recalibrated the age of the Hamilton LF from Whitelaw^173^, which was updated to 4.45±0.04 by García-Navas *et al*.^13^ based on the revised C3n.2n age scaling from Gradstein *et al*.^174^. We therefore use this minimum bound for the divergence of Lagostrophinae, which has been recovered in a more basally branching position relative to Sthenurinae by some phylogenies^3,9,134^. Notably, however, our minimum bound also accommodates for the stratigraphically oldest fossils assigned to the genus *Simosthenurus*, which occur in various localities spanning the lower Pliocene to upper Pleistocene^134^. Nevertheless, unequivocal basally branching sthenurines, such as *Rhizosthenurus flanneryi*, are known from the lower-upper Miocene Riversleigh Zone D^135,165^, which can be constrained at 11.63 Ma using the base of the Tortonian after Cohen *et al*.^149^. The possible referral of *Wanburoo hilarus* to Sthenurinae^3,9^ further extends this range into the middle Miocene Riversleigh Zone C, which we use to delimit our maximum bound of 15.97 Ma from the base of the Langhian^149^.

***Dorcopsis* versus *Dorcopsulus* (crown Dorcopsini)**

Minimum bound. 4.41 Ma.

Maximum bound. 11.63 Ma.

Explanation. The stratigraphically earliest uncontested occurrence of the genus *Dorcopsis* is *Dorcopsis wintercookorum* from the lower Pliocene Hamilton LF of Victoria^175^. García-Navas *et al*.^13^ recalibrated the original radiometric date to a minimum bound of 4.45±0.04. We otherwise set the maximum bound at the base of the Tortonian after Cohen *et al*.^149^ because this accommodates for the upper Miocene (>5.84 Ma^143^) *Dorcopsoides fossilis*, which might either be a sister taxon of *Dorcopsis*, or a more basally branching macropodine^3,9,12,14,26,46,50,53,123–133,135,165^. Furthermore, our maximum fossil age prior is updated from Meredith *et al*.^46^, Dodt *et al*.^118^ and Nilsson *et al*.^119^, all of whom used the absence of demonstrable macropodines in Riversleigh Zone C sites at 16–11.6 Ma^148^.

***Thylogale* versus *Petrogale* + *Dendrolagus* split**

Minimum bound. 4.41 Ma.

Maximum bound. 11.63 Ma.

Explanation. The minimum bound is set at 4.45±0.04 from the lower Pliocene Hamilton LF^13^, which includes *Thylogale ignis*^175^. The maximum bound is designated from the base of the Tortonian after Cohen *et al*.^149^, and delimited by the absence of demonstrable macropodines in Riversleigh Zone C sites^145^ at 16–11.6 Ma^148^.

***Petrogale* versus *Dendrolagus* split**

Minimum bound. 4.41 Ma.

Maximum bound. 11.63 Ma.

Explanation. Meredith *et al*.^46^ set their minimum bound for the divergences of *Petrogale* and *Dendrolagus* at 3.6 Ma; this is equivalent to the latest-early Pliocene Zanclean–Piacenzian boundary after Cohen *et al*.^149^. Eldridge *et al*.^120^ otherwise used 4.46±0.1 to calibrate the *Petrogale*-*Dendrolagus* split based on the minimum age of the lower Pliocene Hamilton LF^176^, which has produced the oldest identified *Dendrolagus* fossils^175^. We accordingly set our minimum bound at 4.45±0.04 based on the Hamilton LF recalibration proposed by García-Navas *et al*.^13^.

Eldridge *et al*.^120^ cited a maximum “radiometric date” of 14.22 Ma based on the lower-middle Miocene Bullock Creek LF of the Northern Territory^177^, and the middle Miocene Riversleigh Zone C^148^, which “records no presence of our ingroup taxa”. While we agree that no crown group macropodine has yet been identified from strata of this age, we prefer to designate our maximum bound at the base of the Tortonian after Cohen *et al*.^149^, which accommodates for the Riversleigh Zone C sites^145^ at 16–11.6 Ma^148^.

***Protemnodon* versus *Macropus+Notamacropus*+*Osphranter* split**

Minimum bound. 4.41 Ma.

Maximum bound. 11.63 Ma.

Explanation. Dawson^140^ concluded that the oldest demonstrable occurrence of *Protemnodon* was in the lower Pliocene Hamilton LF. We therefore use this constraint for our minimum bound of 4.45±0.04 following García-Navas *et al*.^13^. Our maximum bound is designated from the base of the Tortonian after Cohen *et al*.^149^.

***Notamacropus* versus *Osphranter* split**

Minimum bound. 4.41 Ma.

Maximum bound. 11.63 Ma.

Explanation. Our minimum bound is set at 4.45±0.04 based on the revised C3n.2n age scaling of Gradstein *et al*.^174^ for the lower Pliocene Hamilton LF, which includes *Notamacropus* sp.^175^. The maximum bound is designated from the base of the Tortonian after Cohen *et al*.^149^.

**Supplementary references**

1. Johnson, C. N. & Prideaux, G. J. Extinctions of herbivorous mammals in the late Pleistocene of Australia in relation to their feeding ecology: No evidence for environmental change as a cause of extinction. *Austral Ecol.* **29**, 553–557 (2004).
2. Kitchener, D. J. & Friend, J. A. in *The Mammals of Australia* 301–302 (New Holland Publishers, 2008).
3. Helgen, K. M. & Flannery, T. F. Taxonomy and historical distribution of the wallaby genus *Lagostrophus*. *Aust. J. Zool.* **51**, 199–212 (2003).
4. Prince, R. I. T. & Richards, J. D. in *The Mammals of Australia* 406–408 (New Holland Publishers, 2008).
5. Flannery, T. F. *Mammals of the South-West Pacific & Moluccan Islands* (Cornell University Press, 1995).
6. Heinsohn, T. E. Den sites and habitats utilised by the Northern common cuscus *Phalanger orientalis* (Marsupialia: Phalangeridae) in East Timor. *Aust. Mammal*. **27**, 99–101 (2005).
7. Harris, J. M. *Cercartetus concinnus* (Diprotodontia: Burramyidae). *Mammalogy* **831**, 1–11 (2009).
8. Johnson, P. M. & Strahan, R. A further description of the Musky Rat-Kangaroo, *Hypsiprymnodon moschatus* Ramsay, 1876 (Marsupialia, Potoroidae), with notes on its biology. *Aust. Zool.* **21**, 27–46 (1982).
9. Dennis, A. J. & Johnson, P. M. in *The Mammals of Australia* 281–283 (New Holland Publishers, 2008).
10. Dennis, A. J. & Johnson, P. M. in *The Mammals of Australia* 285–286 (New Holland Publishers, 2008).
11. Sinclair, E. A., Danks, A. & Wayne, A. F. Rediscovery of Gilbert’s potoroo, *Potorous tridactylus*, in Western Australia. *Aust. Mammal.* **19**, 69–72 (1996).
12. Green, K., Mitchell, A. T. & Tennant, P. Home range and microhabitat use by the long-footed potoroo, *Potorous longipes*. *Wildl. Res.* **25**, 357–372 (1998).
13. Menkhorst, P. W. & Seebeck, J. H. in *The Mammals of Australia* 299–300 (New Holland Publishers, 2008).
14. Norton, M. A., French, K. O. & Claridge, A. W. Habitat associations of the long-nosed potoroo (*Potorous tridactylus*) at multiple spatial scales. *Aust. J. Zool.* **58**, 303–316 (2010).
15. Frankham, G. J., Handasyde, K. A. & Eldridge, M. D. B. Evolutionary and contemporary responses to habitat fragmentation detected in a mesic zone marsupial, the long-nosed potoroo (*Potorous tridactylus*) in south-eastern Australia. *J. Biogeogr*. **43**, 65 –665 (2016).
16. Flannery, T. F., Martin, R. & Szalay, A. *Tree Kangaroos. A Curious Natural History* (Reed, 1996).
17. Ingleby, S. Distribution and status of the Spectacled Hare-wallaby, *Lagorchestes conspicillatus*. *Wildl. Res.* **18**, 501–519 (1991).
18. Burbidge, A. A. & Johnson, P. M. in *The Mammals of Australia* 314–316 (New Holland Publishers, 2008).
19. Short, J. & Turner, B. The distribution and abundance of the banded and rufous hare-wallabies, *Lagostrophus fasciatus* and *Lagorchestes hirsutus*. *Biol. Conserv.* **60**, 157–166 (1992).
20. Coulson, G. Habitat separation in the Grey kangaroos, *Macropus* *giganteus* Shaw and *M. fuliginosus* (Desmarest) (Marsupialia: Macropodidae), in the Grampians National Park, Western Victoria. *Aust. Mammal.* **13**, 33–40 (1990).
21. Coulson, G. in *The Mammals of Australia* 333–334 (New Holland Publishers, 2008).
22. Coulson, G. in *The Mammals of Australia* 335–337 (New Holland Publishers, 2008).
23. Hinds, L. A. in *The Mammals of Australia* 330–332 (New Holland Publishers, 2008).
24. Maynes, G. in *The Mammals of Australia* 341–342 (New Holland Publishers, 2008).
25. Press, A. J. in *Kangaroos, Wallabies and Rat-kangaroos* 783–786 (Surrey Beatty & Sons, 1989).
26. Tefler, W. R. & Calaby, J. H. in *The Mammals of Australia* 327–328 (New Holland Publishers, 2008).
27. Ingleby, S. & Gordon, G. in *The Mammals of Australia* 359–361 (New Holland Publishers, 2008).
28. Clancy, T. F. & Croft, D. B. in *The Mammals of Australia* 346–348 (New Holland Publishers, 2008).
29. Tefler, W.R & Griffiths, A. D. Dry-season use of space, habitats and shelters by the short-eared rock-wallaby (*Petrogale brachyotis*) in the monsoon tropics. *Wildl. Res.* **33**, 207–214 (2006).
30. Copley, P. B. Studies on the Yellow-footed rock-wallaby, *Petroglae xanthopus* Gray (Marsupialia: Macropodidae). 1. Distribution in South Australia. *Wildl. Res.* **10**, 47–61 (1983).
31. Lim, T. L. & Giles, J. R. Studies on the Yellow-footed rock-wallaby, *Petroglae xanthopus* Gray (Marsupialia: Macropodidae). 3. Distribution and management in western New South Wales. *Wildl. Res.* **14**, 147–161 (1987).
32. Hayward, M. W., de Tores, P. J. & Banks, P. B. Habitat use of the Quokka, *Setonix brachyurus* (Macropodidae: Marsupialia), in the northern Jarrah forest of Australia. *J. Mammal* **86**, 683–688 (2005).
33. Le Mar K. & McArthur, C. Comparison of habitat selection by two sympatric macropods, *Thylogale billardierii* and *Macropus rufogriseus rufogriseus*, in a patchy eucalypt-forestry environment. *Austral Ecol.* **30**, 674–683 (2005).
34. Johnson, K. A. & Rose, R. W. in *The Mammals of Australia* 395–397 (New Holland Publishers, 2008).
35. Merchant, J. C. in *The Mammals of Australia* 404–405 (New Holland Publishers, 2008).
36. Dodt, W. G., Gallus, S., Phillips, M. J. & Nilsson, M. A. Resolving kangaroo phylogeny and overcoming retrotransposon ascertainment bias. *Sci. Rep*. **7**, 16811 (2017).
37. Nilsson, M. A., Zheng, Y., Kumar, V., Phillips, M. J. & Janke, A. Speciation generates mosaic genomes in kangaroos. Genome Biol. Evol. **10**, 33–44 (2018).
38. Eldridge, M. D. B., Beck, R. M. D., Croft, D. A., Travouillon, K. J. & Fox, B. J. An emerging consensus in the evolution, phylogeny, and systematics of marsupials and their fossil relatives (Metatheria). *J. Mammal*. **100**, 802–837 (2019).
39. Lee, M. S. Y. Stability in meaning and content of taxon names: an evaluation of crown-clade definitions. *Proc. R. Soc. Lond. B* **263**, 1103–1109 (1996).
40. Case, J. A. A new genus of Potoroinae (Marsupialia: Macropodidae) from the Miocene Ngapakaldi Local Fauna, South Australia, and a definition of the Potoroinae. *J. Paleontol*. **58**, 1074–1086 (1984).
41. Cooke, B. N. & Kear, B. P. Evolution and diversity of kangaroos (Macropodoidea, Marsupialia). *Aust. Mammal.* **21**, 27–29 (1999).
42. Kear, B. P., Cooke, B. N., Archer, M. & Flannery, T. F. Implications of a new species of the Oligo-Miocene kangaroo (Marsupialia: Macropodoidea) *Nambaroo*, from the Riversleigh World Heritage Area, Queensland, Australia. *J. Paleontol*. **81**, 1147–1167 (2007).
43. Black, K. H., Travouillon, K. J., Den Boer, W., Kear, B. P., Cooke, B. N. & Archer, M. A new species of the basal “kangaroo” *Balbaroo* and a re-evaluation of stem macropodiform interrelationships. *PLoS ONE* **9**, e112705 (2014).
44. Butler, K., Travouillon, K. J., Price, G., Archer, M. & Hand, S. J. Revision of Oligo-Miocene kangaroos, *Ganawamaya* and *Nambaroo* (Marsupialia: Macropodiformes, Balbaridae). *Palaeontol. Electron*. **21.1.8A**, 1–58 (2018).
45. Kear, B. P. & Pledge, N. S. A new fossil kangaroo from the Oligocene-Miocene Etadunna Formation of Ngama Quarry, Lake Palankarinna, South Australia. *Aust. J. Zool.* **55**, 331–339 (2008).
46. Travouillon, K. J., Cooke, B. N., Archer, M. & Hand, S. J. Revision of basal macropodids from the Riversleigh World Heritage Area with descriptions of new material of *Ganguroo bilamina* Cooke, 1997 and a new species. *Palaeontol. Electron*. **17**, 20A (2014).
47. Cooke, B. N., Travouillon, K. J., Archer, M. & Hand, S. J. *Ganguroo robustiter*, sp. nov. (Macropodoidea, Marsupialia), a middle to early late Miocene basal macropodid from Riversleigh World Heritage Area, Australia, *J. Vertebr. Paleontol*. **35**, e956879 (2015).
48. Travouillon, K. J., Archer, M. & Hand, S. J. Revision of *Wabularoo*, an early macropodid kangaroo from mid-Cenozoic deposits of the Riversleigh World Heritage Area, Queensland, Australia. *Alcheringa* **39**, 274–286 (2015).
49. Butler, K., Travouillon, K. J., Price, G. J., Archer, M. & Hand, S. J. *Cookeroo*, a new genus of fossil kangaroo (Marsupialia, Macropodidae) from the Oligo-Miocene of Riversleigh, northwestern Queensland, Australia, *J. Vertebr. Paleontol*. **36**, e1083029 (2016).
50. Travouillon, K. J., Butler, K., Archer, M. & Hand, S. J. New material of *Gumardee pascuali* Flannery *et al*., 1983 (Marsupialia: Macropodiformes) and two new species from the Riversleigh World Heritage Area, Queensland, Australia. *Mem. Mus. Vic*. **74**, 189–207 (2016).
51. Bates, H., Travouillon, K. J., Cooke, B. N., Beck, R. M. D., Hand, S. J. & Archer, M. Three new Miocene species of musky rat-kangaroos (Hypsiprymnodontidae, Macropodoidea): description, phylogenetics and paleoecology. *J. Vertebr. Paleontol*. **34**, 383–396 (2014).
52. Prideaux, G. J. Systematics and evolution of the sthenurine kangaroos. *Univ. Calif. Publ. Geol. Sci.* **146**, 1–623 (2004).
53. Kear, B. P. Phylogenetic implications of macropodid (Marsupialia: Macropodoidea) postcranial remains from Miocene deposits of Riversleigh, northwestern Queensland. *Alcheringa* **26**, 299–318 (2002).
54. Prideaux, G. J. & Tedford, R. H. *Tjukuru wellsi*, gen. et sp. nov., a lagostrophine kangaroo (Diprotodontia, Macropodidae) from the Pliocene (Tirarian) of northern South Australia. *J. Vertebr. Paleontol*. **32**, 717–721 (2012).
55. Flannery, T. F. & Archer, M. Revision of the genus *Troposodon* Bartholomai (Macropodidae: Marsupialia). *Alcheringa* **7**, 263–279 (1983).
56. Potter, S., Cooper, S. J. B., Metcalfe, C. J., Taggart, D. A. & Eldridge, M. D. B. Phylogenetic relationships of rock-wallabies, *Petrogale* (Marsupialia: Macropodidae) and their biogeographic history within Australia. *Mol. Phylogenet. Evol*. **62**, 640–652 (2012).
57. Dawson, L. A new fossil genus of forest wallaby (Marsupialia, Macropodinae) and a review of *Protemnodon* from eastern Australia and New Guinea. *Alcheringa* **28**, 275–290 (2004).
58. Dawson, L., Muirhead, J. & Wroe, S. The Big Sink Local Fauna: a lower Pliocene mammalian fauna from the Wellington Caves complex, Wellington, New South Wales. *Rec. W. Aust. Mus.* **57**, S265–S290 (1999).
59. Warburton, N. M. & Prideaux, G. J. The skeleton of *Congruus kitcheneri*, a semiarboreal kangaroo from the Pleistocene of southern Australia. *R. Soc. Open Sci.* **8,** 202216 (2021).
60. Woodburne, M. O., Macfadden, B. J., Case, J. A., Springer, M. S., Pledge, N. S., Power, J. D., Woodburne, J. M. & Springer, K. B. Land mammal biostratigraphy and magnetostratigraphy of the Etadunna Formation (late Oligocene) of South Australia. *J. Vertebr. Paleontol.* **13**, 483–515 (1993–1994).
61. Megirian, D., Prideaux, G. J., Murray, P. F. & Smit, N. An Australian land mammal age biochronological scheme. *Paleobiol.* **36**, 658–671 (2010).
62. Murray, P. F. & Megirian, D. The Pwerte Marnte Marnte Local Fauna: a new vertebrate assemblage of presumed Oligocene age from the Northern Territory of Australia. *Alcheringa* **30**, S211–S228 (2006).
63. Archer, M. *et al*. Current status of species level representation in faunas from selected fossil localities in the Riversleigh World Heritage Area, northwestern Queensland. *Alcheringa* **30**, S1–S17 (2006).
64. Arena, D. A. *et al*. Mammalian lineages and the biostratigraphy and biochronology of Cenozoic faunas from the Riversleigh World Heritage Area, Australia. *Lethaia* **49**, 43–60 (2016).
65. Archer, M. *et al*. The *Burramys* Project: a conservationist’s reach should exceed history’s grasp, or what is the fossil record for? *Phil. Trans. R. Soc. B* **374**, 20190221 (2019).
66. Woodhead, J. *et al*. Developing a radiometrically-dated chronologic sequence for Neogene biotic change in Australia, from the Riversleigh World Heritage Area of Queensland. *Gond. Res*. **29**, 153–167 (2014).
67. Cohen, K. M., Finney, S. C., Gibbard, P. L. & Fan, J.-X. The ICS International Chronostratigraphic Chart. *Episodes* **36**, 199–204 (2013: updated v.2021/10).
68. Meredith, R. W., Westerman, M. & Springer, M. S. A phylogeny of Diprotodontia (Marsupialia) based on sequences for five nuclear genes. *Mol. Phylogenet. Evol.* **51**, 554–571 (2009).
69. Godthelp, H., Archer, M., Cifelli, R. L., Hand, S. J. & Gilkeson, C. F. Earliest known Australian Tertiary mammal fauna. *Nature* **356**, 514–516 (1992).
70. Meredith, R. W., Westerman, M., Case, J. A. & Springer, M. S. A phylogeny and timescale for marsupial evolution based on sequences for five nuclear genes. *J. Mammal. Evol.* **15**, 1–36 (2008).
71. Beck, R. M. D. A dated phylogeny of marsupials using a molecular supermatrix and multiple fossil constraints. *J. Mammal*. **89**, 175–189 (2008).
72. Hocknull, S. A. Ecological succession during the late Cainozoic of central eastern Queensland: Extinction of a diverse rainforest community. *Mem. Queensl. Mus*. **51**, 39–122 (2005).
73. Brammall, J. & Archer, M*.* Living and extinct petaurids, acrobatids, tarsipedids and burramyids (Marsupialia): relationships and diversity through time. *Aust. Mammal.* **21**, 24–25 (1999).
74. Pledge, N. in *Possums and Opossums: Studies in Evolution* 725–728 (Surrey Beatty and Sons and the Royal Zoological Society of New South Wales, 1987).
75. Brammall, J. & Archer, M. An Oligo-Miocene species of *Burramys* (Marsupialia, Burramyidae) from Riversleigh, northwestern Queensland. *Mem. Queensl. Mus.* **41**, 247–268 (1997).
76. Crosby, K, Godthelp, H., Archer, M. & Pledge, N*.* Diversity and evolution of phalangerid, ektopodontid, miralinid and pilkipildrid marsupials. *Aust. Mammal.* **21**, 22–23 (1999).
77. Crosby, K. Rediagnosis of the fossil species assigned to *Strigocuscus* (Marsupialia, Phalangeridae), with description of a new genus and three new species. *Alcheringa* **31**, 33-58 (2007).
78. Case, J. A., Meredith, R. W. & Person, J. **A pre-Neogene phalangerid possum from South Australia.** *Mus. N. Arizona Bull*. **65**, 659–675 (2009).
79. Flannery, T., Archer, M. & Plane, M. Middle Miocene kangaroos (Macropodoidea: Marsupialia) from three localities in northern Australia, with a description of two new subfamilies. *Bull. Bur. Mineral Resour. Geol. Geophys. Aust.* **7**, 287–302 (1983).
80. Flannery, T. & Rich, T. H. Macropodoids from the middle Miocene Namba Formation, South Australia, and the homology of some dental structures in kangaroos. *J. Paleontol.* **60**, 418–447 (1986).
81. Woodburne, M. O. *Wakiewakie lawsoni*, a new genus and species of Potoroinae (Marsupialia: Macropodidae) of medial Miocene Age, South Australia. *J. Paleontol.* **58**, 1062–1073 (1984).
82. Travouillon, K. J., Archer, M., Hand, S. J. & Godthelp, H. Multivariate analyses of Cenozoic mammalian faunas from Riversleigh, northwestern Queensland. *Alcheringa* **30**, S323–S349 (2006).
83. Kear, B. P., Archer, M. & Flannery, T. F. Bulungamayine (Marsupialia: Macropodoidea) postcranial elements from the late Miocene of Riversleigh northwestern Queensland. *Mem. Assoc. Australas. Palaeontol.* **25**, 103–122 (2001).
84. Woodburne, M. O. The Alcoota Fauna, Central Australia. *Bull. Bur. Mineral Resour. Geol. Geophys. Aust.* **87**, 1–187 (1967).
85. Yates, A. M. & Worthy, T. H. A diminutive species of emu (Casuariidae: Dromaiinae) from the late Miocene of the Northern Territory, Australia. *J. Vertebr. Paleontol*. **39**, e1665057 (2019).
86. Murray, P. The sthenurine affinity of the late Miocene kangaroo *Hadronomus puckridgi* Woodburne 1967 (Marsupialia, Maeropodidae). *Alcheringa* 15, 255–283 (1991).
87. Cooke, B. N. *Wanburoo hilarus* gen et sp. nov., a lophodont bulungamayine kangaroo (Marsupialia: Macropodoidea: Bulungamayinae) from the Miocene deposits of Riversleigh, northwestern Queensland. *Rec. W. Aust. Mus.* **57**, S239–S253 (1999).
88. Pledge, N. S. The Curramulka local fauna: A new late Tertiary fossil assemblage from Yorke Peninsula, South Australia. *The Beagle* **9**, 115–142 (1992).
89. Tedford, R. H. Succession of Pliocene through medial Pleistocene mammal faunas of southeastern Australia. *Rec. S. Aust. Mus*. **27**, 79–93 (1994).
90. Marshall, I. G. Fossil vertebrate faunas from the Lake Victoria region, S.W. New South Wales, Australia. *Mem. Nat. Mus. Vic*. **34**, 151–172 (1973).
91. Whitelaw, M. J. Magnetic polarity stratigraphy of Pliocene and Pleistocene fossil vertebrate localities in southeastern Australia. *Geol. Soc. Am. Bull*. **103**, 1493-1503 (1991).
92. Gradstein, F. M., Ogg, J. G., Schmitz, M. D. & Ogg, G. M. eds. *The Geological Time Scale 2012* (two volumes) (Elsevier, 2012).
93. Flannery, T. F., Rich, T. H. V., Turnbull, W. D. & Lundelius, E. L. Jr. The Macropodoidea (Marsupialia) of the early Pliocene Hamilton Local Fauna, Victoria, Australia. *Fieldiana Geol.* **25**, 1–37 (1992).
94. Turnbull, W. D., Lundelius, E. L. Jr & Archer, M. Dasyurids, perameloids, phalangeroids, and vombatoids from the Early Pliocene Hamilton Fauna, Victoria, Australia. *Bull. Amer. Mus. Nat. Hist.* **279**, 513–540 (2003).
95. Schwartz, L. R. S. A revised faunal list and geological setting for Bullock Creek, a Camfieldian site from the Northern Territory of Australia. *Mem. Mus. Vic*. **74**, 263–290 (2016).
